# Restoration of lipid homeostasis between TG and PE by the LXRα-ATGL/EPT1 axis ameliorates hepatosteatosis

**DOI:** 10.1038/s41419-023-05613-6

**Published:** 2023-02-06

**Authors:** Yulian Chen, Huanguo Jiang, Zhikun Zhan, Jindi Lu, Tanwei Gu, Ping Yu, Weimin Liang, Xi Zhang, Shuwen Liu, Huichang Bi, Shilong Zhong, Lan Tang

**Affiliations:** 1 NMPA Key Laboratory for Research and Evaluation of Drug Metabolism, Guangdong Provincial Key Laboratory of New Drug Screening, School of Pharmaceutical Sciences, Southern Medical University, 510515 Guangzhou, China; 2 Department of Pharmacy, Guangdong Provincial People’s Hospital (Guangdong Academy of Medical Sciences), Southern Medical University, Guangzhou, China

**Keywords:** Dyslipidaemias, Obesity

## Abstract

Converting lipid disturbances in response to energy oversupply into healthy lipid homeostasis is a promising therapy to alleviate hepatosteatosis. Our clinical studies found that a further elevation of triglyceride (TG) in obese patients with the body mass index (BMI) greater than 28 was accompanied by a further reduction of phosphatidylethanolamine (PE). Shorter survival and poor prognosis were shown for the patients with high TG and low PE levels. Liver X receptor alpha (*LXRα*) knockout mice aggravated high-fat diet (HFD)-induced obesity and lipid disorders, making the TG enrichment and the PE decrease more pronounced according to the liver lipidomics analysis. The RNA-seq from mice liver exhibited that these metabolism disorders were attributed to the decline of *Atgl* (encoding the TG metabolism enzyme ATGL) and *Ept1* (encoding the PE synthesis enzyme EPT1) expression. Mechanistic studies uncovered that LXRα activated the *ATGL* and *EPT1* gene via direct binding to a LXR response element (LXRE) in the promoter. Moreover, both the supplement of PE in statin or fibrate therapy, and the LXRα inducer (oridonin) ameliorated cellular lipid deposition and lipotoxicity. Altogether, restoration of lipid homeostasis of TG and PE via the LXRα-ATGL/EPT1 axis may be a potential approach for the management of hepatosteatosis and metabolic syndrome.

## Facts

- Lipidomics data from clinical patients and mice indicated obesity causes lipid disturbances, especially between TG and PE;
- LXRα knockout mice exacerbated lipid disturbance of TG and PE;
- TG metabolizing enzyme ATGL and phospholipid synthase EPT1 were regulated by LXRα;
- Restoring the homeostasis of TG and PE via LXRα-ATGL/EPT1 axis alleviated hepatosteatosis.

## Introduction

Triglycerides (TGs) store energy as neutral lipids during times of energy oversupply and serve as an energy reservoir during deprivation [1]. Phosphatidylethanolamine (PE) is the second most abundant phospholipid in mammals, providing critical structural support for cell membranes and contributing to the formation and stabilization of membrane protein [2–5]. Recent studies indicated that PE reduction is accompanied by the TG accumulation leading to the exacerbation of a series of metabolic diseases [3, 6, 7]. To this end, the synthesis, catabolism, and transformation of glycerolipids affects many cellular processes critical for lipid homeostasis, prompting us to explore the complex relationship between lipid homeostasis and obesity treatment [8–10].

TG is decomposed into diglyceride (DG), monoacylglycerol, and free FFA by adipose triglyceride lipase (ATGL) and hormone-sensitive lipase (HSL) in sequence [11]. FFAs are necessary for both energy supply and thermogenesis [12, 13]. However, FFA supply from the circulation precisely matches mitochondrial capacity for oxidative metabolism in healthy lipid homeostasis. Enhanced lipolytic activity releases toxic FFAs, which cause cellular lipotoxicity [8, 14–16]. As the secondary decomposition products of TGs, DGs are a direct precursor for phospholipid synthesis in the Kennedy pathway, which is a major route for PE synthesis in mammalian cells [17]. The transfer of phosphoethanolamine from CDP-ethanolamine to DG is catalyzed by Ethanolamine phosphotransferase 1 (EPT1) to synthesize PE [18]. Therefore, we speculated that rerouting of TG decomposition products into PE synthesis can solve the problem of the lipotoxicity caused by active lipolysis [14, 15].

Altered lipid partitioning at the molecular level leads to disruption of lipid homeostasis. PLA2 hydrolyzes membrane phospholipids to generate fatty acids, which promotes TG synthesis [19]. PCYT2 catalyzes the rate-controlling step in the Kennedy pathway for the synthesis of PE. Elimination of PCYT2 abolishes the utilization of DG by the Kennedy pathway, resulting in increased TG in hepatocytes [9]. After acute nerve injury, decreasing TG synthesis and enhancing phospholipid synthesis through the Kennedy pathway promoted axon regeneration [10]. It shows that lipid disturbances and lipotoxicity in vivo are closely related to phospholipid decomposition and TG synthesis.

It remains to be studied that modulation of nuclear transcription factors may antagonize lipotoxicity by controlling downstream functional genes to bidirectionally regulate TG metabolism and phospholipid synthesis. Liver X receptors (LXRs) include the two isoforms called LXRα and LXRβ [20]. LXR activation promotes the transcriptional activation of the cholesterol efflux transporters (ABCA1 and ABCG1) and APOE to mediate the efflux of extra cholesterol [21]. Activated LXR led to the transcriptional induction of the insulin-sensitive glucose transporter (GLUT4), and improves insulin resistance [22]. When LXR activates, Lpcat3 promotes the incorporation of unsaturated FFAs into PL and ameliorates ER stress and inflammation [23]. In general, LXRs are key regulators of lipid homeostasis and effective inhibitors of inflammation [24–26]. However, the function of promoting lipolysis and remodeling excess TG broken products into phospholipid synthesis in vivo to reduce cellular lipotoxicity, especially maintaining lipid homeostasis by the downstream mechanism of LXRα, has not yet been explored.

Here, this study aimed to investigate whether restoration of lipid homeostasis between TG and PE via LXRα-ATGL/EPT1 axis has a critical role in alleviating steatosis and lipotoxicity. Lipidomics data showed that obesity causes the disturbance of TG and PE in vivo. Mechanistic studies uncovered that LXRα deficiency, which reduces the TG metabolizing enzyme ATGL and the phospholipid synthase EPT1, is the main molecular mechanism leading to the imbalance between TG and PE. Moreover, TG accumulation and lipotoxicity could be decreased by supplementing with appropriate amounts of PE or using natural agonizts of LXRα (oridonin). These observations outline an essential role for the LXRα-ATGL/EPT1 axis in the coordination between TG and PE. Restoring lipid homeostasis in TG and PE may be beneficial to hepatosteatosis. These notions provide new insights for the treatment of metabolic diseases induced by excess fat intake.

## Results

### Lipid disturbances were associated with the elevation of TG and the reduction of PE

**Fig. 1:**
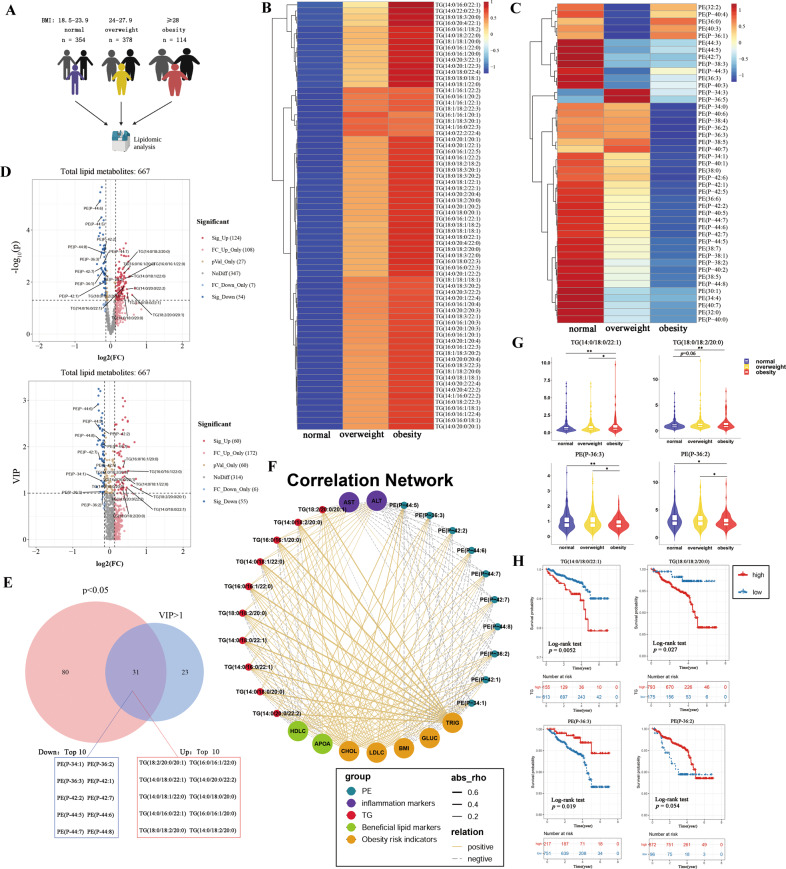
Lipid disturbances were associated with the elevation of TG and the reduction of PE. **A** Experiment schematic for lipidomic analyses grouped by BMI according to the Chinese reference standard. **B** Heatmap of TG species containing different lengths of fatty acids in the three groups of patient plasma samples of normal, overweight, and obesity. The increased TG species code the color as red in the heatmap; the decreased ones code blue. **C** Heatmap of PE species containing different lengths of fatty acids in the three groups of patient plasma samples of normal, overweight and obesity. The increased PE species code the color as red in the heatmap; the decreased ones code the blue. **D** The volcano plots showing the differential lipid metabolite level between group normal and obesity. **E** Venn diagram showing the overlapping and accession-specific differential lipid metabolites. Combined with VIP value (VIP > 1) and *P* value (*P* < 0.05), the top ten PE and TG lipid metabolites with the most significant differences have been listed, and then marked in volcano plots. **F** Correlation network among the biochemical indicators and the top ten PE and TG lipid metabolites with the most significant differences. APOA apolipoprotein A, HDLC high-density lipoprotein cholesterol, ALT alanine aminotransferase, AST aspartate aminotransferase, LDLC low-density lipoprotein cholesterol, BMI body mass index, GLUC blood glucose, CHOL cholesterol, TRIG triglyceride. **G** Violin plot of individual TG and PE species included in the list of the top ten lipid metabolites with the most significant differences. The width of the graph represents the distribution probability of the *y* axis value, reflecting the overall distribution of the data. *P < 0.05; **P < 0.01. **H** The TG or PE species shown in the violin plot were divided into a high (red) and low (blue) group according to the content, and Kaplan–Meier analysis was performed to compare survival for patients with high (red) and low (blue) TG or PE species. The surv_cutpoint function determines the best cut point for continuous variables (log-rank test, *P* values were displayed in the corner). See also Supplementary Fig. S1.

Lipidomics were performed on plasma of 1011 human patients and 667 lipid metabolites were measured to identify the lipid profiles altered between the lean and obese. Referring to the BMI classification of Chinese adults [27, 28], 866 patients with valid parameters were randomly divided into three groups: BMI 18.5–23.9 was normal (*n* = 354); BMI 24–27.9 was overweight (*n* = 378), and BMI ≥ 28 (*n* = 114) was obesity (Fig. 1A). As the grades of normal, overweight, and obesity increased, neutral lipids gradually increased and phospholipids decreased (Supplementary Fig. S1A, B). Detailed analysis of lipid classes with different FFA composition revealed that the obesity group specifically displayed higher TG levels and lower PE levels (Fig. 1B, C), both of which were abundant in neutral lipids and phospholipids, respectively [1, 29].

We further performed differential metabolite screening among the normal and the obesity group based on the independent samples *t* test (*P* < 0.05) and variables identified as important in the projection (VIP) (VIP > 1) scores. The screening results were presented as volcano plots (Fig. 1D). For the detected PE and TG metabolites, the top ten most differential TGs and PEs of lipid metabolites were listed from the overlapping part of the Venn diagram, respectively, and marked in the volcano plot for subsequent analysis (Fig. 1D, E). Next, correlation analysis was performed to determine the potential association of lipid metabolite abundance with human plasma biochemical indicators (Fig. 1F, Supplementary Fig. S1C, and Supplementary Table S3). We observed that the ten differential TGs which were enriched in the obesity group were positively correlated while the PEs, which were depleted in the obesity group, were negatively correlated with obesity risk indicators (BMI, GLUC, TRIG) and inflammation markers (AST, ALT) (Fig. 1F). Moreover, the ten TGs were negatively correlated while the PEs were positively correlated with beneficial lipid markers (HDLC, APOA) (Fig. 1F). In particular, the relative levels of TG(14:0/18:0/22:1) and TG(18:0/18:2/20:0) were sequentially increased, when the relative levels of PE(P-36:3) and PE(P-36:2) were sequentially reduced from normal to overweight, and to obesity (Fig. 1G). By dividing 968 patients with valid parameters into two groups based on the content of TGs and PEs, the Kaplan–Meier plotting showed shorter survival and a poorer prognosis for the high TG(14:0/18:0/22:1) and TG(18:0/18:2/20:0) levels, while longer survival and better prognosis for high PE(P-36:3) and PE(P-36:2) levels (Fig. 1H). Overall, these indicate that elevated TG and reduced PE may be important contributors to hepatosteatosis.

### *LXRα* deficiency aggravates HFD-induced obesity

**Fig. 2:**
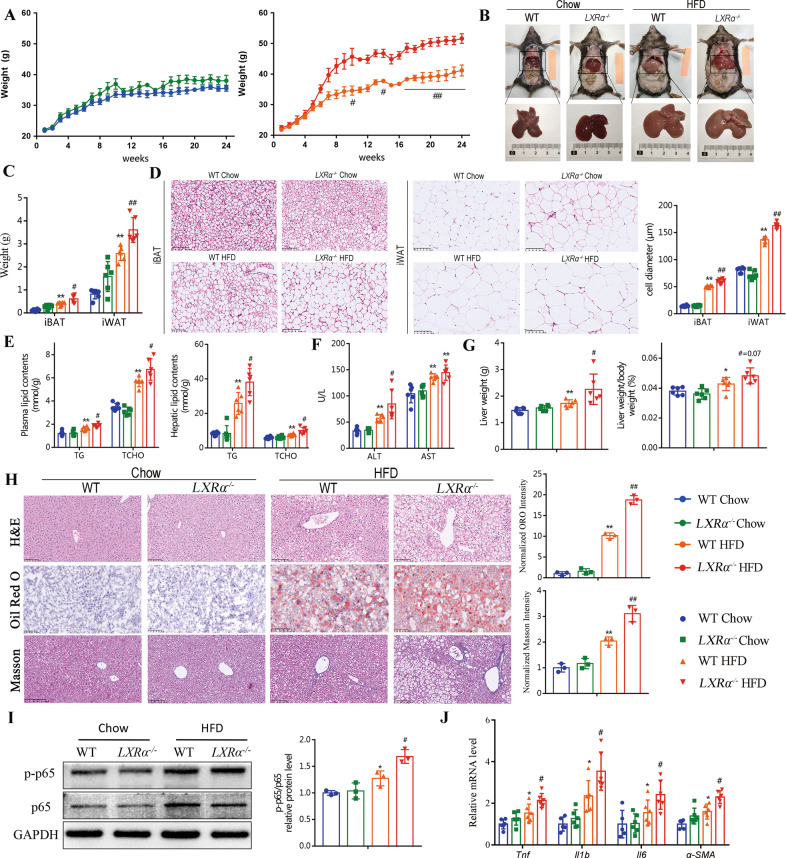
*LXRα* deficiency aggravates HFD-induced obesity and related comorbidities. Mice were randomly assigned to four groups: WT Chow, *LXRα*^−/−^ Chow, WT HFD, *LXRα*^−/−^ HFD, and used for the following assays: **A** Bodyweight (weekly) (*n* = 6). **B** Representative liver and adipose tissue photos. **C** Weight of iBAT and iWAT (*n* = 6). **D** Representative images of H&E staining of iBAT and iWAT. Adipocyte diameter was calculated. Scale bar, 100 µm. **E** Plasma and hepatic triglyceride (TG) and cholesterol (CHOL) levels (*n* = 6). **F** Plasma ALT and AST levels (*n* = 6); **G** Liver weight and liver weight-to-bodyweight ratios (*n* = 6). **H** Representative H&E, Oil Red O (ORO), Masson’s trichrome, and insert of ORO and Masson intensity (*n* = 3); Scale bar, 100 μm. **I** p65 phosphorylation levels in the livers of mice (*n* = 3). **J** mRNA levels of genes associated with inflammatory response (*n* = 6). Data are presented as mean ± SD. *P < 0.05 and **P < 0.01 were compared with WT Chow; ^#^*P* < 0.05 was compared with WT HFD.

LXRα plays a critical role in regulating lipid homeostasis and inflammation [24–26]. We investigated the role of LXRα in lipid homeostasis by feeding *LXRα*^−/−^ mice and their control wild-type mice (WT) with normal chow or HFD for 24 weeks (Supplementary Fig. S3A). *LXRα* ablation had no effect on the bodyweight of mice fed a chow diet (Fig. 2A). However, *LXRα*^−/−^ mice gained 25% more bodyweight than controls after a 24-week HFD feeding (Fig. 2A), although the energy intake was similar between mice with different genotypes (Supplementary Fig. S3B). Accordingly, in the anatomical map, HFD-fed *LXRα*^−/−^ mice showed more lipid deposition and an enlarged liver (Fig. 2B). Supporting this, the weight of interscapular brown adipose tissue (iBAT) and inguinal white adipose tissue (iWAT) in HFD-fed *LXRα*^−/−^ mice were significantly increased by 49% and 39%, respectively, compared to those in WT mice fed with the same diet (Fig. 2C). Histological analysis revealed dramatically adipocyte hypertrophy in HFD-fed *LXRα*^−/−^ mice (Fig. 2D). And adipocytes in iBAT of HFD-fed *LXRα*^−/−^ mice were 18% larger than those in WT mice, and 19% larger in iWAT (Fig. 2D).

After the HFD feeding period, *LXRα*^−/−^ mice had higher levels of triglyceride (TG) and cholesterol (CHOL) in both plasma and liver (Fig. 2E). Meanwhile, the elevated plasma ALT, liver weight and the ratio of liver weight to bodyweight were aggravated in *LXRα*^-/-^ mice (Fig. 2F, G). Histological analysis of liver sections was imaged and quantified more severe hepatocyte injury (ballooning), lobular inflammation, steatosis and hepatic fibrosis in *LXRα*^−/−^ HFD group compared to WT HFD group (Fig. 2H). HFD-induced p65 phosphorylation further enhanced in *LXRα*^−/−^ mice (Fig. 2I). The hepatic mRNA levels of genes associated with proinflammatory factors and fibrosis were higher in HFD-fed *LXRα*^−/−^ mice than in WT mice with the same diet (Fig. 2J). The ELISA result of Il1b was consistent with the mRNA result (Supplementary Fig. S3C). Besides, their glucose tolerance, as well as insulin sensitivity, were both impaired (Supplementary Fig. S3D–F). Taken together, these results suggest that *LXRα* deficiency exacerbates HFD-induced obesity, including hyperlipidemia, hepatic steatosis, hepatic inflammation, and glucose intolerance.

### *LXRα* deficiency exacerbates HFD-induced lipid disorders between TG and PE

**Fig. 3:**
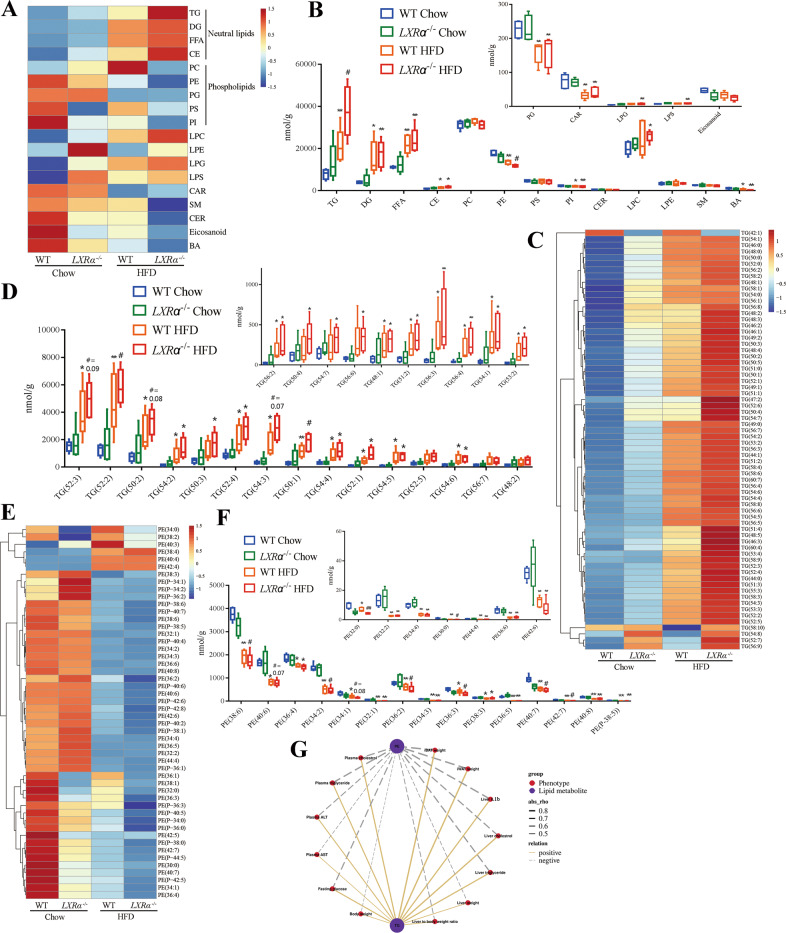
*LXRα* deficiency exacerbates HFD-induced lipid disorders between TG and PE. **A** Heatmap of average levels of lipid species concentrations (nmol/g) in liver tissues (*n* = 5). **B** Amount of lipid species in liver tissues illustrated as concentration (nmol/g) of each lipid species. Lipid species with very low concentrations are shown in the right corner of the panel (*n* = 5). **C** Heatmap of species of TG containing different lengths of fatty acids based on the concentrations (nmol/g) in liver tissues. The increased TG species code the color as the red in the heatmap; the decreased ones code the blue (*n* = 5). **D** Abundant and representative concentration (nmol/g) of TG containing different lengths of fatty acids in the liver tissues; TG species with very low concentration are shown in the right corner of the panel (*n* = 5). **E** Heatmap of species of PE containing different lengths of fatty acids based on the concentrations (nmol/g) in liver tissues. The increased PE species code the color as red in the heatmap; the decreased ones code the blue (*n* = 5). **F** Abundant and representative concentration (nmol/g) of PE containing different lengths of fatty acids in the liver tissues; PE species with very low concentration are shown in the right corner of the panel (*n* = 5). *P < 0.05 and **P < 0.01 were compared with WT Chow; ^#^*P* < 0.05 was compared with WT HFD. **G** Correlation network among the mice phenotypes and the lipid metabolites of total TG and PE.

We investigated how the loss of *LXRα* modulated lipid synthesis and metabolism in the HFD. We performed lipidomic analysis of mice livers from group WT Chow, *LXRα*^−/−^ Chow, WT HFD and *LXRα*^−/−^ HFD (*n* = 5/each cohort). The alterations of neutral lipids and phospholipids were less remarkable between group WT Chow and *LXRα*^−/−^ Chow, and more profound between normal chow diet and HFD (Fig. 3A, B). Among the major neutral lipids, the TG level in group WT HFD was 155% higher than that in group WT Chow, and highest in group *LXRα*^−/−^ HFD (increased 80% compared to group WT HFD) (Fig. 3B). In contrast, the PE level in group WT HFD was 22% less than that in group WT Chow, and least in group *LXRα*^−/−^ HFD (reduced 14% compared to group WT HFD) (Fig. 3B). Principal coordinate analysis (PCA) and OPLS-DA models were carried out to indicate that group WT Chow clearly separated from WT HFD, and WT HFD clearly separated from *LXRα*^−/−^ HFD (Supplementary Fig. S4A, B). It suggested major distinctions in the lipid metabolic profiles were mainly due to the HFD, and *LXRα* deficiency made this difference more obvious (Supplementary Fig. S4A, B).

We thus analyzed the TG composition of the various lipid species (Fig. 3C, D). Similarly, the concentration of the long-chain triglycerides (LCT) such as TG(52:3), TG(52:2), TG(50:2), TG(54:3), TG(50:1), which were the most abundant TG species, were higher in group WT HFD and rose to the highest levels in *LXRα*^−/−^ HFD (Fig. 3C, D). In the case of PE, the levels of PE(38:6), PE(40:6), PE(34:2), and PE(34:1) accounted for a large proportion in PE species, whose content experienced a decline in group WT HFD, and saw a continuous drop in the *LXRα*^−/−^ HFD (Fig. 3E, F). According to the correlation analysis between the lipid metabolites and the mice phenotypes, we observed that total TGs which were enriched in HFD-fed mice were positively correlated while total PEs, which were cut down in HFD-fed mice, were negatively correlated with the obesity risk indicators and inflammation markers, such as plasma cholesterol and triglyceride, plasma ALT and AST, fasting glucose, bodyweight, liver weight and liver to bodyweight ratio, liver cholesterol, and triglyceride, liver Il1b, iBAT and iWAT weight (Fig. 3G, Supplementary Fig. S4C, and Supplementary Table S4). Based on these observations, the results are consistent with the hypothesis of TG enrichment and PE depletion during the occurrence of obesity-induced lipid disorders, which corroborates our findings from human patients to animal experiments. The obesity-induced lipid imbalances exacerbate by *LXRα* deficiency, suggesting that *LXRα* plays an important role in regulating the homeostasis between TG and PE.

### Decrease of *Atgl* and *Ept1* is an important downstream mechanism of lipid disorder in HFD-induced hepatosteatosis

**Fig. 4:**
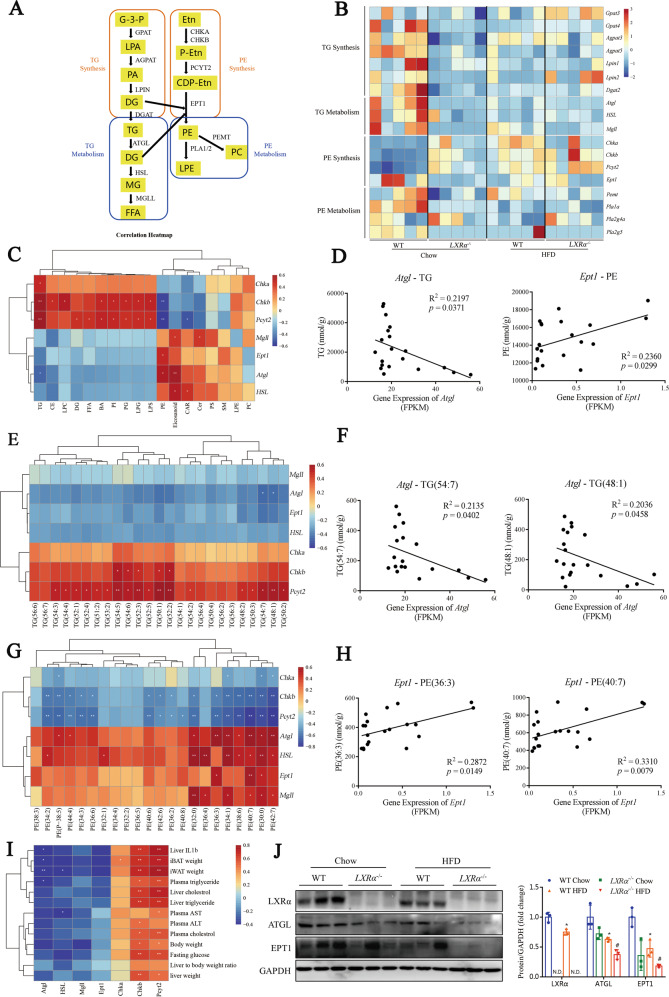
Decrease of *Atgl* and *Ept1* is an important cause of lipid disorders. **A** Investigating genetic mechanisms affecting lipid homeostasis through a working model of lipid homeostasis pathways including glycerolipid metabolism and synthesis process. G-3-P glycerol-3-phosphate, LPA lysophosphatidic acid, PA phosphatidic acid, DG diglyceride, TG triglyceride, FFA free fatty acid, Etn ethanolamine, P-Etn phosphoethanolamine, CDP-Etn CDP-ethanolamine, PE phosphatidylethanolamine, LPE lysophosphatidylethanolamine, PC phosphatidylcholine. **B** Heatmap of genes related to lipid catabolism and synthesis (data obtained from GEO database with an accession number of GSE204986). **C** Correlation heatmap between total lipid metabolites and genes associated with TG metabolism and PE synthesis (*n* = 5 per group). **D** Pearson’s correlation between *Atgl* expression and TG content, and Pearson’s correlation between *Ept1* expression and PE content. **E** Correlation heatmap between species of TG containing different lengths of fatty acids and genes associated with TG metabolism and PE synthesis. **F** Pearson’s correlation between *Atgl* expression and TG(54:7) or PE(48:1) content. **G** Correlation heatmap between species of PE containing different lengths of fatty acids and genes associated with TG metabolism and PE synthesis. **H** Pearson’s correlation between *Ept1* expression and PE(36:3) or PE(40:7) content. **I** Correlation heatmap between mice phenotypes and genes associated with TG metabolism and PE synthesis. **J** Western blot analysis of LXRα, ATGL and EPT1 proteins in mice from group WT Chow, *LXRα*^−/−^ Chow, WT HFD and *LXRα*^-/−^ HFD (*n* = 3 per group). Data are presented as mean ± SD. *P < 0.05 were compared with WT Chow; ^#^*P* < 0.05 was compared with WT HFD.

Here, we focused on exploring the molecular mechanism by which the homeostasis of TG and PE is disrupted in HFD-induced obesity. We showed the synthesis and decomposition pathway of TG and PE in vivo, and RNA-seq was performed to display the genes involved in the pathway as a heatmap (Fig. 4A, B, GSE204986). Most of the correlation coefficients of gene expression among samples in the same group were greater than 0.85, indicating good biological repeatability (Supplementary Fig. S5A). The analysis revealed that the suppressed expression of genes for TG metabolism and PE synthesis was responsible for the results of TG accumulation and PE depletion in HFD-fed and *LXRα*-deficient mice, while changes in genes of TG synthesis and PE metabolism did not correspond to the lipidomics results (Fig. 4A, B). Correlation analysis was performed to determine the potential association of gene expression for TG metabolism and PE synthesis with lipid classes (Fig. 4C). We found that the expression of the TG hydrolysis gene, *Atgl*, was negatively correlated with TG (*P* = 0.0371), while the expression of PE synthesis gene, *Ept1*, was positively correlated with PE (*P* = 0.0299) (Fig. 4C, D).

Next, the detailed analyses were confirmed the correlation between the lipid species with various FFA composition and genes for TG decomposition and PE synthesis. Likewise, *Atgl* expression correlated negatively with TGs with various FFA composition (Fig. 4E, F), while *Ept1* expression correlated positively with PEs with various FFA composition (Fig. 4G, H). In addition, *Atgl* and *Ept1* expression were both negatively correlated with mice phenotypes of obesity risk indicators and inflammation markers (Fig. 4I). Analyses of the gene matrix from the GEO Profiles database (Reference Series: GDS4830, GDS4881) showed that *LXRα, EPT1* and *ATGL* expression levels were attenuated in HFD-fed mice or in clinical patients with disrupted insulin sensitivity (Supplementary Fig. S5B, C), which corroborated our discoveries from previous analyses. HFD-induced obesity decreased the protein expression of ATGL and EPT1, while LXRα deficiency further suppressed their expression (Fig. 4J). These support the notion that the decrease of EPT1 and ATGL is an important downstream mechanism of lipid disorder in HFD-induced hepatosteatosis.

### LXRα plays an essential role in maintaining lipid homeostasis by affecting TG metabolism and PE synthesis

We wondered whether restoring LXRα expression in HFD-induced obesity could preserve lipid homeostasis by up-regulating ATGL and EPT1 expression. The experimental mice were divided into six groups, including WT Chow, WT HFD, WT GW, *LXRα*^−/−^ Chow, *LXRα*^−/−^ HFD, and *LXRα*^−/−^ GW (Supplementary Fig. S6A). The obvious separation trend from PCA and OPLS-DA score plot indicated metabolic differences for mice among WT GW, WT HFD and *LXRα*^−/−^ GW group (Supplementary Fig. S6B, C).

**Fig. 5:**
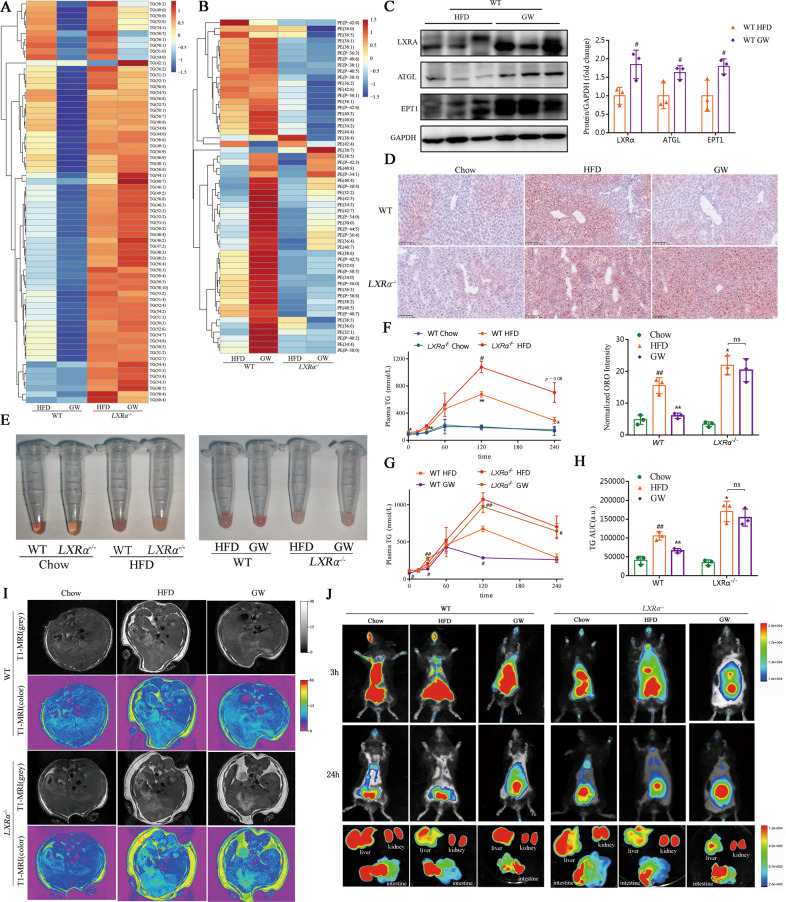
LXRα plays an important role in maintaining lipid homeostasis by affecting TG metabolism and PE synthesis. **A** Heatmap of species of TG containing different lengths of fatty acids based on the concentrations (nmol/g) in the liver of mice treated with LXR agonizts GW3965 or not. The increased TG species code the color as red in the heatmap; the decreased ones code the blue (*n* = 5). **B** Heatmap of species of PE containing different lengths of fatty acids based on the concentrations (nmol/g) in the liver of mice treated with vehicle or GW3965. The increased PE species code the color as red in the heatmap; the decreased ones code the blue (*n* = 5). **C** Western blot analysis of LXRα, ATGL and EPT1 proteins in mice from group WT HFD, WT GW. **D**–**H** Image of Oil red O staining of liver of mice (Insert showing the ORO intensity) (**D**), plasma samples (**E**) and plasma TG levels (inset showing the AUC values for plasma TG) (**F**–**H**) after oral gavage of olive oil (10 μl/g) (*n* = 3). Data are mean ± SD. *P < 0.05 and **P < 0.01 were compared with WT Chow; ^#^*P* < 0.05 and ^##^*P* < 0.01 were compared with WT HFD. The samples shown in plasma photos and ORO staining were collected at 2 h after oral gavage of olive oil. Scale bar, 100 μm. **I** Representative T1-MRI images showing lipid accumulating contents in livers of mice treated with vehicle or GW3965. **J** Representative in vivo imaging system images of mice after tail vein injection of cy7-labeled phosphoethanolamine. And representative images of cy7-labeled phosphoethanolamine fluorescence intensity in mice live. Mice livers, kidneys, and intestines were collected at 24 h after tail vein injection of cy7-labeled phosphoethanolamine.

We compared the total lipidomic profile of TG and PE in four groups: WT HFD, WT GW, *LXRα*^−/−^ HFD and *LXRα*^−/−^ GW (Supplementary Fig. S6D). In contrast to mice in WT HFD group, mice in WT GW group received a HFD could attenuate TG accumulation and increase PE production, but there was no alteration between group *LXRα*^−/−^ HFD and *LXRα*^−/−^ GW (Fig. 5A, B). GW3965 treatment potentiated the expression of ATGL and EPT1 (Fig. 5C). According to the results of olive oil gavage experiment and magnetic resonance imaging (MRI), mice in *LXRα*^−/−^ HFD group exhibited higher circulating TG levels, increased serum lactescence, more serious hepatic lipid droplet deposition and broader subcutaneous fat distribution compared to those in the WT HFD group (Fig. 5D–I). In addition, in vivo tracking experiments with cy7-labeled phosphoethanolamine and cy7-labeled cholesterol were indicated that *LXRα*^−/−^ mice fed a HFD showed lower phosphoethanolamine utilization and cholesterol efflux efficiency (Fig. 5J and Supplementary Fig. S6E, F). These results suggested that *LXRα* deficiency resulted in lower rates of TG catabolism and PE production (Fig. 5D–J and Supplementary Fig. S6E, F). In contrast, the LXRα agonist, GW3965, showed the opposite effects that could alleviate the lipid disturbances attributed to the HFD (Fig. 5D–J and Supplementary Fig. S6E, F). These findings support the role of LXRα in maintaining lipid homeostasis by affecting TG metabolism and PE synthesis in a HFD.

### LXRα regulates the transcription of *EPT1* and *ATGL*

**Fig. 6:**
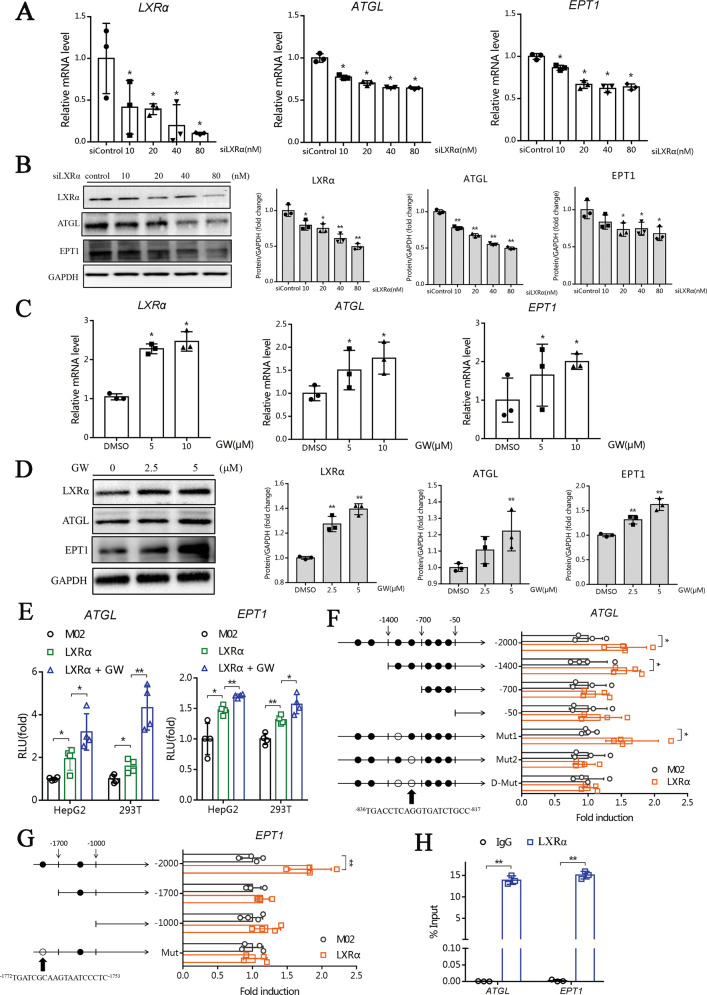
LXRα regulates the transcription of *EPT1* and *ATGL*. **A** *LXRα*、*ATGL* and *EPT1* mRNA levels in HepG2 cells transfected with siLXRα or siControl (*n* = 3). **B** LXRα, ATGL, and EPT1 protein levels in HepG2 cells transfected with siLXRα or siControl (*n* = 3). **C** *LXRα*、*ATGL*、*EPT1* mRNA levels in HepG2 cells treated with DMSO or GW3965 (*n* = 3). **D** LXRα, ATGL, and EPT1 protein levels in HepG2 cells treated with DMSO or GW3965 (*n* = 3). *P < 0.05 and **P < 0.01 were compared with control (siControl, DMSO). **E** LXRα induced *ATGL* and *EPT1* transcription in luciferase reporter assays. HepG2 cells and 293T cells were transfected with *ATGL*-luciferase reporter or *EPT1*-luciferase reporter and LXRα plasmid. After 24 h of transfection, 5 μM GW3965 or DMSO treatment was performed and luciferase reporter activities were measured (*n* = 4). **F**–**G** Effects of LXRα on the activities of various versions of *ATGL*-luciferase or *EPT1*-luciferase reporters (*n* = 4). **H** ChIP assays showing significant recruitment of LXRα protein to the LXRE located on the promoter region of *ATGL* and *EPT1* (*n* = 3). Data are mean ± SD. *P < 0.05; **P < 0.01.

The changes of EPT1 and ATGL correspond to the results of lipidomics, which prompted us to investigate whether LXRα regulates EPT1 and ATGL using an activation mechanism. The siLXRα dose-dependently inhibited the mRNA and protein expression of ATGL and EPT1 in HepG2 cells (Fig. 6A, B), whereas LXRα agonist GW3965 treatment resulted in increased expression of ATGL and EPT1 in HepG2 cells (Fig. 6C, D). In luciferase reporter assays, LXRα induced the promoter activity of *ATGL* and *EPT1* (Fig. 6E). JASPAR predicted the transcription factor binding site (TFBS) information of LXRα protein binding to the *ATGL* and *EPT1* promoter (Supplementary Fig. S7A). ChIP-seq data from Cistrome DB suggested the enrichment of LXRα protein to *ATGL* and *EPT1* promoter (Cistrome DB ID: 69801, GEO ID: GSM2042848) (Supplementary Fig. S7B). Sequence analysis indicated seven and two potential LXR response elements (LXREs) (i.e., the putative DNA motifs for LXRα binding and action) in the promoter regions of *ATGL* and *EPT1* genes, respectively (Fig. 6F, G). Truncation and mutation experiments identified the LXREs in the *ATGL* (−836/−817 bp) and *EPT1* (−1772/−1753 bp) promoters, which was actually responsible for LXRα action (Fig. 6F, G). Moreover, chromatin immunoprecipitation (ChIP) assays revealed significant recruitment of LXRα protein to the LXRE of *ATGL* and *EPT1* (Fig. 6H). Taken together, LXRα drives the transcription of *ATGL* and *EPT1* through direct binding to the LXRE in the gene promoters.

### Promoting TG hydrolysis and PE supplementation ameliorates lipid accumulation and lipotoxicity in primary hepatocytes from *LXRα*^−/−^ mice

**Fig. 7:**
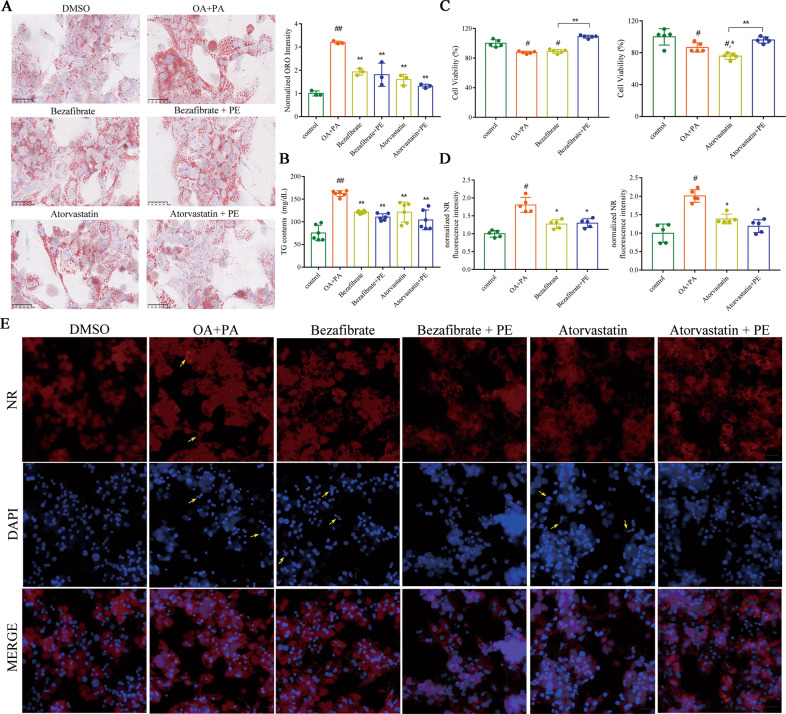
Promoting TG hydrolysis and PE supplementation ameliorate lipid accumulation and lipotoxicity in primary hepatocytes from *LXRα*^−/−^ mice. Primary hepatocytes from *LXRα*^−/−^ mice were treated with hypolipidemic drugs (Atorvastatin or Bezafibrate) in response to control solvent or OA + PA (OA, 500 μM; PA, 250 μM) stimulation for 48 h, and used for the following assays: **A** Images and quantitation of Oil red O staining (*n* = 3); scale bar, 50 μm; **B** TG contents in primary hepatocytes from *LXRα*^−/−^ mice. Data are mean ± SD (*n* = 6). ^#^*P* < 0.05 and ^##^*P* < 0.01 were compared with the control (DMSO) group. *P < 0.05 and **P < 0.01 were compared with OA + PA group. **C** CCK8 assay to access cell viability; Data are mean ± SD (*n* = 5). ^#^*P* < 0.05 and ^##^*P* < 0.01 were compared with the control (DMSO) group. *P < 0.05 and **P < 0.01 were compared with OA + PA group. **D** Normalized Nile Red (NR) fluorescence intensity. Data are mean ± SD (*n* = 5). ^#^*P* < 0.05 and ^##^*P* < 0.01 were compared with control (DMSO) group. *P < 0.05 and **P < 0.01 were compared with OA + PA grou*p*. The higher the fluorescence intensity value, the higher the lipid level in the cell. **E** The neutral lipid location (NR staining) and apoptotic nuclei morphology changes (DAPI staining) in primary hepatocytes from *LXRα*^-/-^ mice (*n* = 3); scale bar, 100 μm.

When statin or fibrate is employed to lower blood lipids for the treatment of cardiovascular diseases, there was reported that an increased risk of myalgia, diabetes mellitus, and hepatic transaminase elevated [30]. Our study found a decline of PE in obesity-induced lipid disorders. To this end, we wondered whether additional PE supplementation in conventional lipid-lowering drug therapy could alleviate lipotoxicity. Here, primary hepatocytes extracted from *LXRα*^−/−^ mice were treated with palmitic acid (PA) (0.25 mM) and oleic acid (OA) (0.5 mM) for 48 h to induce deposition of lipid droplets. These cells were simultaneously treated with a series of concentrations of PE in the presence of PA and OA. We monitored that OA and PA stimulation was able to significantly lower the cell viability (Fig. 7A). PE treatment at 5 μM could increase the cell viability by 17%, and 10 μM by 21% (Supplementary Fig. S8). Oil Red O (ORO) staining showed that cellular lipid droplet accumulation was notably decreased by Atorvastatin and Bezafibrate, and the combined treatment with PE did not weaken the lipid-lowering effect (Fig. 7A). Similar results were obtained when TG contents and Nile Red (NR) fluorescence intensity were directly detected and quantified (Fig. 7B, D). The CCK8 assay indicated that when Atorvastatin and Bezafibrate treatment failed to restore normal cell viability, the combined treatment of 10 μM PE could significantly improve cell viability (Fig. 7C). The combination of NR staining and DAPI staining could further verify the above results. Lipid-lowering effect of Atorvastatin and Bezafibrate was imaged by NR staining (Fig. 7E). Furthermore, apoptotic nuclei morphological changes were still observed after Atorvastatin or Bezafibrate treatment, but disappeared after combined treatment with 10 μM PE (Fig. 7E). Altogether, we demonstrated that lipid-lowering therapy alone failed to ameliorate lipotoxicity such as apoptosis. Moderate PE treatment may reduce lipotoxicity by protecting cell membranes.

### The natural LXRα small-molecule inducer (oridonin) ameliorates cellular lipid accumulation

**Fig. 8:**
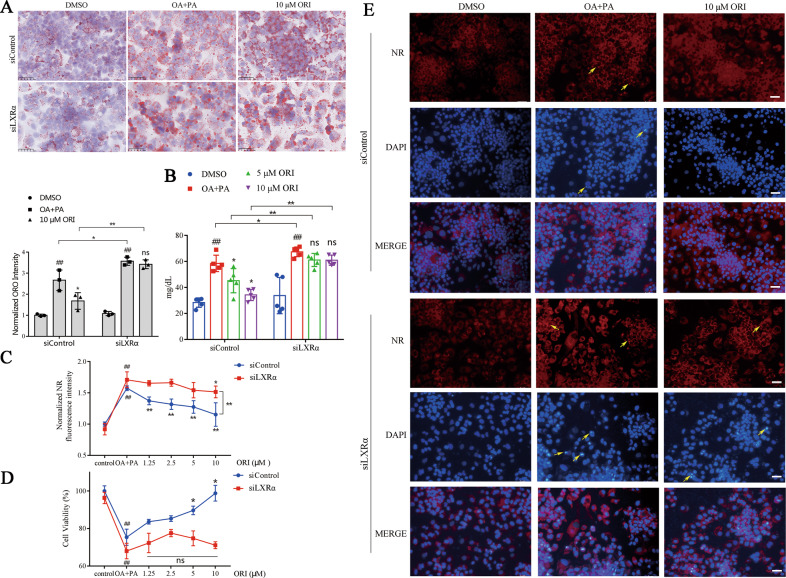
The natural LXRα small-molecule inducer (oridonin) ameliorates cellular lipid accumulation. **A** Oil red O staining of HepG2 cells treated with 10 μM oridonin (ORI) for 48 h in response to OA and PA after transfection with siLXRα or siControl. Scale bar, 50 μm. Data are mean ± SD (*n* = 3). ^#^*P* < 0.05 and ^##^*P* < 0.01 were compared with DMSO group. *P < 0.05 and **P < 0.01 were compared with OA + PA group. **B** TG contents in HepG2 cells treated with ORI after transfection with siLXRα or siControl in response to OA and PA. Data are mean ± SD (*n* = 5). ^#^*P* < 0.05 and ^##^*P* < 0.01 were compared with DMSO group. *P < 0.05 and **P < 0.01 were compared with OA + PA group. **C**, **D** Normalized Nile Red (NR) fluorescence intensity and cell viability in HepG2 cells treated with different concentrations of ORI after transfection with siLXRα or siControl. The higher the fluorescence intensity value, the higher the lipid accumulation level in the cell. Data are mean ± SD (*n* = 5). ^#^*P* < 0.05 and ^##^*P* < 0.01 were compared with DMSO group. *P < 0.05 and **P < 0.01 were compared with OA + PA group. **E** The neutral lipid location (NR staining) and apoptotic nuclei morphology changes (DAPI) in HepG2 cells treated with 10 μM ORI after transfection with siLXRα or siControl in response to OA and PA (*n* = 3). Scale bar, 100 μm.

Given the critical role of LXRα in the development of HFD-induced obesity, it is of interest to test whether LXRα can be targeted to prevent lipid disorder. Therefore, we examined the effects of the small molecule oridonin (ORI) on lipid disorder. ORI has been verified as a LXRα inducer that could potentiate LXRα expression in our previous study [31]. In this study, we demonstrated that ORI could increase the mRNA and protein expression of LXRα, but siLXRα dramatically attenuated this induction of ORI (Supplementary Fig. S9A–C). Afterward, HepG2 cells were transfected with siLXRα and siControl, and then treated with ORI in the presence or absence of 0.25 mM PA and 0.5 mM OA (OA + PA) for 48 h. ORO staining and the cellular TG contents confirmed a reduction of cellular lipid droplet accumulation by ORI (Fig. 8A, B). NR fluorescence intensity and cell viability detection further verified the lipid-lowering effect and cytoprotective effect of ORI (Fig. 8C, D). However, these effects were almost abolished by pretreatment with siLXRα (Fig. 8A–D). In addition, the suppression of lipid deposition and disappearance of abnormal nuclei morphological changes were monitored in the graph of NR staining and DAPI staining by ORI treatment, whose effects were abrogated by the pretreatment with siLXRα (Fig. 8E). These results corroborate that LXRα plays an essential role in protecting lipid homeostasis and indicate that small-molecule drug development targeted restoration of LXRα expression can ameliorate obesity-induced lipid disturbances.

### Restoration of LXRα function alleviates the deleterious effects of LXRα ablation on primary hepatocytes

**Fig. 9:**
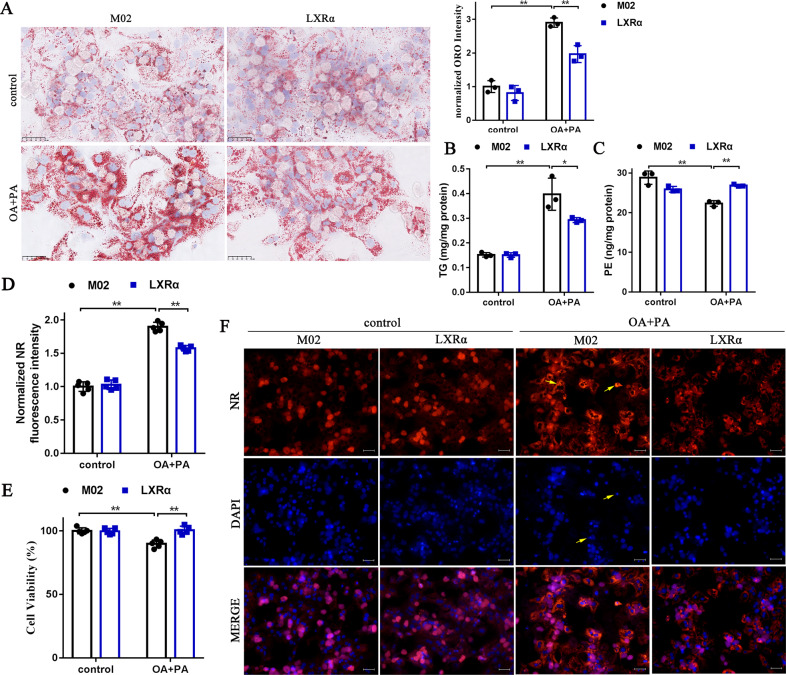
Restoration of LXRα function alleviates the deleterious effects of LXRα ablation on primary hepatocyte. **A** Oil red O staining of primary hepatocyte from LXRα^−/−^ mice in response to control reagent or OA + PA after transfection with M02 or LXRα expression plasmid. Scale bar, 50 μm. Data are mean ± SD (*n* = 3). *P < 0.05; **P < 0.01. **B**, **C** TG and PE contents in hepatocyte from LXRα^−/−^ mice in response to control reagent or OA + PA after transfection with M02 or LXRα expression plasmid. Data are mean ± SD (*n* = 3). *P < 0.05; **P < 0.01. **D**, **E** Normalized Nile Red (NR) fluorescence intensity and cell viability in hepatocyte from LXRα^−/−^ mice in response to control reagent or OA + PA after transfection with M02 or LXRα expression plasmid.　Data are mean ± SD (*n* = 5). *P < 0.05; **P < 0.01. **F** The neutral lipid location (NR staining) and apoptotic nuclei morphology changes (DAPI) in hepatocytes from LXRα^-/-^ mice in response to control reagent or OA + PA after transfection with M02 or LXRα expression plasmid. Scale bar, 100 μm.

Accordingly, to investigate whether restoration of LXRα function alleviates the deleterious effects of LXRα ablation, we compared primary hepatocytes isolated from *LXRα*^−/−^ mice after transfection with M02 or LXRα expression plasmid in the presence of control reagent or OA + PA. The model validations of LXRα overexpression on primary hepatocyte from LXRα^−/−^ mice were revealed in Supplementary Fig. S10. LXRα restoration on LXRα knockdown hepatocytes attenuated the increase of lipid accumulation and TG level (Fig. 9A, B, D), while the decline of PE content and cell viability were abrogated (Fig. 9C, E). In the NR staining and DAPI staining analysis, restoring LXRα function on LXRα knockdown hepatocytes showed the suppression of lipid accumulation and decline of abnormal nuclei morphological changes (Fig. 9F).

## Discussion

In order to preserve a healthy energy supply and lipid homeostasis, the synthesis and metabolism of glycerolipids form a balanced circulatory system in vivo. Obesity and its complications result in a progressive disruption of lipid homeostasis [24, 32]. In this paper, we clarified the functions of LXRα-ATGL/EPT1 axis in maintaining the lipid balance between TG metabolism and PE synthesis and used it in the study for the treatment of obesity-induced lipid disturbance.

Lipidomics data from clinical patient plasma demonstrated the TG enrichment and the PE depletion in obesity patients (Fig. 1), which supports our working hypotheses. Besides, a drop of PE in Drosophila stimulates aberrant activation of the sterol regulatory element-binding protein (SREBP) pathway leading to increased TG accumulation [6]. The absence of PE synthesis by the Kennedy pathway disrupted skeletal muscle lipid homeostasis leading to accumulation of both TG and DG [3]. Furthermore, a global decrease of phospholipids were observed in nonalcoholic steatohepatitis (NASH) patients [7]. In a word, these studies seem to be consistent with the results that the disruption of the balance of TG and PE is associated with obesity and related metabolic syndromes.

Several pharmacological strategies for hepatic and metabolic disorders, including the modulation of nuclear transcription factors, are at the preclinical or early clinical stages of development [33–35]. For instance, the LXR nuclear receptors are crucial transcription factors acting as important mediators, whose target genes encode proteins that have critical roles in modulating either cholesterol homeostasis via sterol response element-binding protein (SREBP-1c), cholesterol efflux transporters (ABCA1) and murine 7A-hydrox-ylase (CYP7A1) or glucose homeostasis via GLUT4 [21–23]. It is reported that LXRα governs tissue-specific lipoprotein lipase (LPL) and hormone-sensitive lipase (HSL) expression, expanding its role as key regulator of lipid degradation [36, 37]. Furthermore, LPCAT3 as a direct target gene of LXRα is required for the maintenance of the lipid bilayer of the cell membrane and crucial for its functions [21, 23]. Whereas, the comprehensive balance and functions maintained by LXRα in different classes of lipids is rarely explored.

In this study, we have shown that *LXRα*^−/−^ mice aggravated steatosis and lipid metabolism disorder especially between TG and PE in the case of HFD feeding (Figs. 2 and 3). A screen of major genes responsible for the biochemical process of TG and PE homeostasis from RNA-seq identified *Atgl* and *Ept1* as the potential linkers of LXRα with TG metabolism and PE synthesis (Figs. 4 and 5). Direct regulation of *ATGL* and *EPT1* by LXRα was validated by cell-based assay (Fig. 6). However, Homozygous *Atgl* mice targeted mutation have defects in lipolysis and changed energy metabolism. Triglyceride accumulation in cardiac muscle leads to severe cardiac dysfunction and mortality [38]. Homozygous *Ept1* mice deletion exhibit early-stage embryonic lethality prior, with a rare survival to the perinatal stage [39]. These reports limit our further study of lipid alteration in vivo after *Atgl* and *Ept1* ablation using knockout mice.

Our study proposed a therapeutic potential to administer an appropriate amount of PE as a cytoprotective agent while enhancing TG breakdown in obesity treatment (Fig. 7). PE synthesis is an important physiological activity that modulates the balance between TG as energy storage and FFA as energy expenditure depot, protecting a healthy lipid balance in vivo [10, 40–42]. A hepatoprotective drug developed by supplementing endogenous phospholipids, polyene phosphatidylcholine has been explored to be effective and economical for patients with liver disease [43]. TG is an abundant neutral lipid that functions as a storage depot for toxic FFAs [1]. But in the case of obesity, it often occurs that adipose tissue exceeds its lipid storage capacity and TG non-physiologically accumulates in hepatocytes leading to lipotoxicity [1, 32, 44]. PE is required for the formation of cell membranes and organelle membranes, which is involved in anti-inflammatory and cell surface attachment functions [18]. Our work pointed out that part of TG is decomposed into FFAs for oxidative metabolism and energy utilization, and the other part is decomposed into secondary product DG remodeling into phospholipids in vivo, most likely as an effective therapy to transform the lipid disorder of energy oversupply into healthy lipid homeostasis.

Transcriptional control by a nuclear receptor is a complex process and is regulated at multiple levels [21, 45, 46]. The perception of LXRα should develop into a broader view of its role on lipid biosynthesis and metabolism in combination with its anti-inflammatory and cell membrane structure-regulating effects, positioning it as an important player in protecting lipid homeostasis [21, 47, 48]. Our study showed that ORI is an effective natural LXRα inducer [31]. Cell experiments demonstrated that ORI can simultaneously reduce lipid accumulation, protect cell viability and reduce lipotoxicity by activating LXRα (Fig. 8). Furthermore, LXRα restoration alleviates the deleterious effects of LXRα ablation on primary hepatocytes (Fig. 9). These results imply a comprehensive role for LXRα in maintaining lipid homeostasis.

**Fig. 10:**
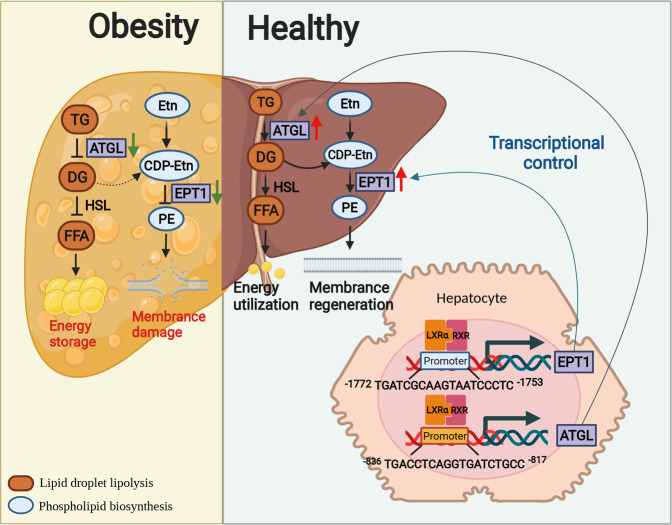
Mechanism illustration. The schematic presentation depicts the proposed mechanism by which restoration of lipid homeostasis between TG and PE via the LXRα-ATGL/EPT1 axis ameliorates obesity and related complications.

In summary, lipid homeostasis, especially the balance of TG and PE, is disrupted in the case of excess dietary fat. We revealed that the regulation of lipid homeostasis between TG and PE by LXRα functions to prevent hepatosteatosis. LXRα regulates dynamic homeostasis between TG and PE via promoting *ATGL* and *EPT1* transcription and expression. These findings suggest that restoring the balance between TG and PE via the LXRα-ATGL/EPT1 axis may be a viable therapeutic strategy for hepatosteatosis (Fig. 10).

## Materials and methods

### Materials

The primary antibodies for western blotting were as follows: anti-phospho-NF-κB (p-p65) antibody (1:1000 dilution, 3033T, CST, USA), anti-NF-κB (p65) antibody (1:1000 dilution, 8242T, CST, USA), anti-LXRα antibody (1:500 dilution, ab41902, ab176323, Abcam, USA), anti-EPT1 antibody (1:250 dilution, ab194554), anti-ATGL antibody (1:500 dilution, DF7756, Affinity, China), anti-GAPDH antibody (1:3000 dilution, ab8345), HRP-conjugated goat anti-rabbit IgG (1:3000 dilution, ab6721), HRP-conjugated goat anti-mouse IgG (1:3000 dilution, ab6789). LXRα antibody (61175) for ChIP assay was purchased from Active Motif (USA). Cy7-labeled phosphoethanolamine and cy7-labeled cholesterol were purchased from Xi’an Qiyue Biotechnology Co., Ltd. The siRNA specific for LXRα (sc-38828), and negative control siRNA (sc-37007) were obtained from Santa Cruz Biotechnology (Dallas, TX, USA). The liver X receptor agonist GW3965 was purchased from Selleck (Shanghai, China). Oridonin was purchased from Aladdin (28957-04-2, Shanghai, China). Tnf (A104732) and Il1b (A105903) ELISA kits for mouse liver tissue were obtained from Shanghai Fusheng Industrial Co. (Shanghai, China). LXRα plasmids (EX-A1306-M02, EX-Mm19582-M02) were purchased from GeneCopoeia Inc. (Guangzhou, China). *ATGL* and EPT1-luciferase reports (*ATGL*: −2000/+200, −1400/+200, −700/+200, −50/+200; *EPT1*: −2000/+200, −1700/+200, −1000/+200, and mutated version) were obtained from Transheep (Shanghai, China). Mouse liver PE Elisa kit (TW12464) was purchased from Shanghai Tongwei Industry Co.

### Animals

Wild-type C57BL/6 mice were purchased from the laboratory animal center of Southern Medical University (Guangzhou, Guangdong, China). *LXRα*^−/−^ mice (KOCMP-21253-Nr1h3) have been established by the CRISPR/Cas9 system and validated Cyagen Biosciences (Guangzhou) Inc. (Guangzhou, Guangdong, China). The mice were identified by PCR genotyping and expression profiling (Supplementary Fig. S2). Genotyping of mice was performed with primers F1: 5’-CTGCAACCAACACCAGTCTTCAATC-3’, R1: 5’-CTAAAGCAAGAATGAAGGCCACTGC-3’ and F2: 5’-CTCTGCAATCGAGGTGGCTGGAAAG-3’, resulting in a 656 bp fragment for homozygotes and a 521-bp fragment for the wild-type allele. *LXRα* knockout (*LXRα*^−/−^) mice were identified by PCR genotyping of genomic DNA from tail biopsies and had both wild-type allele and heterozygotes (Supplementary Fig. S2A). The mice showed the predicted loss of LXRα expression in the liver with RT-qPCR, western blot analysis and IGV visualization of high-throughput sequencing (Supplementary Fig. S2B–D). All mice were housed in pathogen-free conditions in a temperature-controlled environment at 22–24 °C with a 12-h/12-h light/dark cycle. All protocols for animal experiments were approved by the Institutional Animal Care and Use Committee of Southern Medical University (Guangzhou, China).

### Clinical samples

The 1011 patients who received percutaneous coronary intervention (PCI) treatment in Guangdong Provincial People’s Hospital from 2010 to 2013 were followed up for all-cause death and major adverse cardiovascular event (MACE) up to 5 years (ClinicalTrials.govID: NCT03797339). All the procedures were approved by the Institutional Review Board of Guangdong Provincial People’s Hospital (Guangzhou, China) (NO.GDREC2010137/2017071H) and conducted in accordance with the Declaration of Helsinki. The patients participating in the study have been informed in detail the purpose and process of the research and signed an informed consent form (nos. 20100910 and 20170211). For plasma samples from patients, the widely targeted lipidomic profiling was performed using UPLC-MS/MS system at Wuhan Metware Biotechnology with the same method as described in Setion Lipidomic profiling. The details of the study populations are obtained in this paper [49].

### Lipidomic profiling

For animal samples, lipid metabolite profiling was carried out using a targeted quantitative lipidomics by Wuhan Metware Biotechnology Co., Ltd. (Wuhan, China). Previously frozen liver tissue (50 mg) was homogenized in 1 ml lipid extract (methyl tert-butyl ether: methanol = 3:1). Tissue samples were whirled in the mixture at 4 °C for 2 min. Then, 200 µl of deionized H_2_O was added to the mixture, followed by centrifuging with 16,000×*g* at 4 °C for 10 min. The extract supernatant was dried and redissolved. Then, a liquid chromatography-electrospray ionization-tandem mass (LC-ESI-MS/MS) system (UPLC, ExionLC™AD, https://sciex.com.cn/; MS, QTRAP® 6500 + , https://sciex.com.cn/) was performed for metabolite quantification. The internal standards were shown in Supplementary Table S2. In brief, the analytical conditions were as follows, UPLC: column [Thermo C30 (2.6 μm, 2.1 mm × 100 mm)], solvent system [A: acetonitrile/water (60/40, v/v) containing 0.1% methanoic acid and 10 mmol/L ammonium formate, B: acetonitrile/isopropanol (10/90, v/v) containing 0.1% methanoic acid and 10 mmol/L ammonium formate], a graded series of A/B program [80:20 (v/v) at 0 min, 70:30 (v/v) at 3.0 min, 40:60 (v/v) at 4 min, 15:85 (v/v) at 9 min, 10:90 (v/v) at 14 min, 5:95 (v/v) at 15.5 min, 5:95 (v/v) at 17.3 min, 80:20 (v/v) at 17.5 min and 80:20 (v/v) at 20 min], flow rate (0.35 ml/min), temperature, 45 °C; injection volume: 2 μl. After the samples were quality controlled, a total of 473 lipid metabolites were detected.

### HFD-induced obesity

To determine the effect of LXRα on lipid homeostasis, *LXRα* knockout (*LXRα*^−/−^) mice and wild-type (WT) mice were fed for 24 weeks at 6–8 weeks of age. In addition, mice were treated with GW3965 (30 mg kg^−1^) or vehicle by oral gavage once three days at the 16th week. The mice in the control group were kept on the chow diet. Bodyweight was recorded weekly during the 24-week experiments. Diets were normal chow (SPF-F02-002, SPF (Beijing) Biotechnology Co., Ltd.) and HFD (60% calories from fat, HF60, Dyets, USA). Anatomical imaging of body fat was monitored using a mouse MRI (PharmaScan70/16) by ast spin-echo T1-weighted.

### GTT and ITT

GTT and ITT were assessed in mice fasted for 12 and 6 h, respectively. For GTT, mice were intraperitoneally injected with 1 g kg^−1^ glucose, while 0.75 IU kg^−1^ insulin was intraperitoneally injected into mice for ITT. Blood glucose was tested before (time 0) and at 15, 30, 60, and 120 min after injection by Handheld blood glucose meter.

### Oil gavage experiment

Mice were gavaged with olive oil (10 μl/g) after overnight fasting. Blood samples were collected by orbital bleeding before (time 0) and at 15, 30, 60, 120, and 240 min after oil gavage. TG were determined with assay kit (A110-1-1, Nanjing Jiancheng Bioengineering Institute, Nanjing, China). In addition, 2 h post gavage, the plasma and liver samples from fasted mice were collected and imaged to access lipid accumulation.

### Bioluminescence imaging

In vivo bioluminescence imaging was performed on isoflurane-anesthetized animals after cy7-labled phosphoethanolamine (6.25 mg kg^−1^) or cy7-labeled cholesterol (10 mg kg^−1^) was injected into tail vein of isoflurane-anesthetized animals. Images were captured using a multimodal small-animal in vivo imaging system (FX Pro, Bruker, USA) with an open filter.

### Transcriptome

Total RNA isolation was performed using the RNAprep Kit (RE-03014, FOREGENE, Chengdu, China) according to the manufacturer’s instructions. Transcriptome libraries were produced and sequenced on the Illumina HiSeq 4000 platform by LC-Bio Technology Co., Ltd (Hangzhou, China). The low-quality reads were filtered out following the quality control procedures and used for the genome assembly. The gene expression levels were assessed as the number of reads per kilobase of gene length per million mapped reads (FPKM). RNA-seq data were obtained from GEO database with an accession number of GSE204986.

### Biochemical analyses

Serum triglyceride (TG), total cholesterol (TC), alanine aminotransferase (ALT), and aspartate aminotransferase(AST) levels were measured by using a Pointcare Automatic biochemical analysis system (S/N:31919, MNCHIP, Tianjin, China) according to the manufacturer’s instructions. Samples were sonicated in ethanol for the determination of TG in animal liver tissue and cells using assay kit (A110-1-1, Jiancheng Bioengineering Institute).

### Histology

The specimens from the liver and adipose tissue were collected and fixed with 4% paraformaldehyde solution (Solarbio), dehydrated, and embedded in paraffin. In all, 6–10-μm paraffin-embedded sections were stained with hematoxylin & eosin (H&E) and Masson’s trichrome.

For Oil Red O staining, liver samples were frozen in −80 °C and embedded in OCT compound. In all, 10-µm-thick sections were prepared and stained with Oil red O (ORO). The above staining followed by counterstaining with hematoxylin. Images were captured using CX-31 microscope (Olympus Corporation, Japan). Adipocyte size was quantified using the NanoZoomer Digital Pathology software and the stained area of the lesion was determined by ImageJ software based on six sections per mouse and six mice per group.

### RT-qPCR

Total RNA was extracted using RNAprep Kit (RE-03014, FOREGENE, Chengdu, China) and reversely transcribed to cDNA using RT Master Mix (RR037A, Takara, Shiga, Japan). RT-qPCR was carried out using SYBR Green Master Mix (A6002, Promega, WI, USA). Data were normalized to the housekeeping gene (*GAPDH*). Primers are listed in Supplementary Table S1.

### Western blot analysis

The tissue and cell samples were lysed in RIPA lysis buffer containing 100× Protease Inhibitor (MB2678, Meilunbio, Dalian, Liaoning). Protein concentrations were detected using a BCA assay kit (P0012S, Beyotime, Shanghai, China). The samples (25 µg) were loaded on 10% SDS‐PAGE gel and then transferred to polyvinylidene fluoride membranes (Millipore, USA) for 1.5 h by wet transfer method. After being blocked with 5% skimmed milk, the membranes were incubated with primary antibody overnight at 4 °C, followed by incubation with HRP-conjugated secondary antibody. The blots were visualized by using FluorChem R system (ProteinSimple, USA). The integrated optical density value of each stripe was calculated by ImageJ Software.

### Isolation of primary hepatocytes and cell culture

HepG2 and 293T cells were cultured in Dulbecco’s modified Eagle’s medium (DMEM) (10270106, Gibico, USA) supplemented with 10% fetal bovine serum (FBS) (FSP500, ExCell Bio, China) at 37 °C in a humidified atmosphere containing 5% CO_2_. Cells were transfected with overexpression plasmid or siRNA using Lipofectamine™ 3000 Transfection Reagent (L3000001, Invitrogen Life Technologies, Carlsbad, USA). After 24–48 h, cells were collected for further analysis.

To mimic the NAFLD model in vivo, HepG2 cells were treated with cell culture medium containing the indicated concentrations of palmitic acid (PA) (0.25 mM) and oleic acid (OA) (0.5 mM) (SYSJ-KJ006, Kunchuang, Xian, China) for 48 h.

Primary hepatocytes were isolated from LXRα^−/−^ mice by liver perfusion. After pentobarbital sodium anesthetized, mice were opened to the peritoneal cavity. Livers were perfused with Hanks’ Balanced Salt Solution (HBSS) (5 mmol L^−1^ CaCl_2_, 0.1 mg mL^−1^ DNaseI, and 100 mmol L^−1^ HEPES) for 5 min through the portal vein, followed by a second perfusion with collagenase buffer (0.35 mg mL^−1^ collagenase, 66.7 mmol L^−1^ NaCl, 6.7 mmol L^−1^ KCl, 50 mmol L^−1^ HEPES, and 4.8 mmol L^−1^ CaCl_2_ for another 5 min. After the livers were gently minced with forceps, the cell suspensions were filtered through a 100-µm nylon cell strainer (SORFA, Zhejiang, China). Then the suspensions were centrifuged at 50×*g* for 2 min and resuspended in DMEM supplemented with 10% FBS and 1% penicillin–streptomycin solution. Finally, cells were incubated at 37 °C for 24 h prior to all experiments.

### Luciferase report assay

HepG2 or 293T cells were co-transfected with ATGL or EPT1-luciferase reporter (300 ng), pRL-nTK (a renilla luciferase reporter, 60 ng) and overexpression LXRα plasmid (600 ng) using Lipofectamine™ 3000 Transfection Reagent. Then, cells were lysed in a passive lysis buffer 24 h later and luciferase activities were measured using Dual-Luciferase Reporter Assay System (E1910, Promega, Madison, USA). Firefly luciferase activity was normalized to Renilla luciferase activity and showed as a relative luciferase unit (RLU).

### ChIP

ChIP assays were performed using a Chromatin IP kit (Axl-ChIP001, Axl-Bio, Guangzhou, China). HepG2 cells were fixed in 1% formaldehyde and lysed in lysis buffer, followed by sonication (UCD-300, Diagenode, Belgium). The chromatin samples were immunoprecipitated with anti-LXRα antibody or normal IgG (a negative control) overnight at 4 °C. The immune complex was de-cross-linked at 65 °C water bath for 6 h. The obtained DNAs were purified and analyzed by qPCR with specific primers (Supplementary Table S1).

### Cell counting kit-8 (CCK8)

The CCK8 reagent (CK04, Dojindo, Japan) was mixed with DMEM (1:10) to prepare the working solution. Subsequently, the supernatant was removed from the cells in a 96-well plate and 110 µl of the working solution was added to incubate at 37 °C for 1 h. Absorbance was measured at 450 nm to assess cell viability.

### Nile red (NR) fluorescence determination and staining with DAPI

NR powder (7385-67-3, Biotopped, Beijing, China) was formulated into a 1 mg/ml stock solution with DMSO. Dilute the stock solution with PBS at a dilution of 1:2000 to prepare a working solution. After the supernatant was removed from the cells, each well of the 96-well plate was added 100 μl of working solution, incubated for 15 min, and washed three times with PBS. After setting the excitation light wavelength of the microplate reader (M1000PRO, TECAN) to 485 nm and the emission wavelength to 580 nm, the fluorescence intensity was measured (the higher the fluorescence intensity value, the higher the lipid level in the cell).

For the NR staining, cells were incubated in 4% paraformaldehyde for 30 min. NR staining was performed as described above. Then, the cell samples were covered in the DAPI (C0065, Solarbio) solution for 5 min. The images were captured using Inverted Fluorescence Microscope (Axio Observer A1, Carl Zeiss, Oberkochen, Germany).

### Statistical analyses

Data are recorded as mean ± standard deviation (SD). Prism 7 software (GraphPad) was used to perform statistical tests. Statistical analysis was performed with Student’s *t* test and two-way analysis of variance (ANOVA). Then, post hoc Bonferroni test was used for further group comparisons if the ANOVA analysis was significant. A combined analysis of multiple omics was performed by Pearson correlation analysis. *P* < 0.05 was considered to be significant (*P < 0.05, **P < 0.01).

## Supplementary information

supporting meterial1

Original Data File

checklist

## Data Availability

The clinical trials number is registered in the U.S. National Library of Medicine (ClinicalTrials.govID: NCT03797339). The RNA-seq datasets generated in this paper are available at NCBI GEO database (GSE204986). The data supporting the findings of this study are available in “Materials and methods” of the article and/or the Supplementary Materials. RNA-seq data were obtained from GEO database with an accession number of GSE204986. Additional supporting data are available from the corresponding authors upon reasonable request.

## Materials availability

This study did not generate new unique reagents.
