# Supplementary material for: Restoration of lipid homeostasis between TG and PE by the LXRα-ATGL/EPT1 axis ameliorates hepatosteatosis
Source: Cell Death Dis. 2023 Feb 6;14(2):85. doi: 10.1038/s41419-023-05613-6 (PMC9902534; doi:10.1038/s41419-023-05613-6)
Supplement: Supplementary file 1 — supporting meterial1 [file 41419_2023_5613_MOESM1_ESM.pdf]

Supplementary Materials for

Restoration of lipid homeostasis between TG and PE by the LXRα-ATGL/EPT1 axis ameliorates obesity

Yulian Chen[1#], Huanguo Jiang[1#], Zhikun Zhan[1#], Jindi Lu[1], Tanwei Gu[1], Ping Yu[1], Weimin Liang[1], Xi Zhang[1], Shuwen Liu[1], Huichang Bi[1], Shilong Zhong[1 2] and Lan Tang[1*]*

[*]Corresponding Author Address: NMPA Key Laboratory for Research and Evaluation of Drug Metabolism, Guangdong Provincial Key Laboratory of New Drug Screening, School of Pharmaceutical Sciences, Southern Medical University, Guangzhou 510515, China. Tel. /fax: +86 20-61648596. Email: Lan Tang, tl405@smu.edu.cn; Shilong Zhong, zhongsl@hotmail.com.

Table S1. Oligonucleotides used in this study.

Gene	Forward (5'-3' sequence)	Reverse (5'-3' sequence)
**qPCR**		
human-*LXRα*	GCTGCAAGTGGAATTCATCAACC	ATATGTGTGCTGCAGCCTCTCCA
human-*EPT1*	TGGCGCCCAATCTGATAACT	GTTCTGCGAGCTTGCTTTCC
human-*ATGL*	ACCAGCATCCAGTTCAACCT	ATCCCTGCTTGCACATCTCT
human-*GAPDH*	GCTCTCTGCTCCTCCTGTTC	ACGACCAAATCCGTTGACTC
mice-*Il1b*	GAAATGCCACCTTTTGACAGTG	TGGATGCTCTCATCAGGACAG
mice-*Tnf*	GACGTGGAACTGGCAGAAGAG	TTGGTGGTTTGTGAGTGTGAG
mice-*Il*6	TAGTCCTTCCTACCCCAATTTCC	TTGGTCCTTAGCCACTCCTTC
mice-*α-SMA*	GACGTACAACTGGTATTGTG	TCAGGATCTTCATGAGGTAG
mice-*Gapdh*	AGGTCGGTGTGAACGGATTTG	TGTAGACCATGTAGTTGAGGTCA
**ChIP**		
ATGL-LXRE	CCCGAGTAGCTAGGACTACAT	GTGGCCCATGCCTGTAAT
EPT1-LXRE	TTGAGACGAGGTTTCACCATATT	CTCACGCTTGTAATCCCTACAC

Table S2. The UPLC-MS/MS methodology for quantification of lipid metabolite profiling.

Lipid (abbreviation)	Common Name	Internal Standard	Precursor m/z	Product 1 m/z	Ionization	ION_MODE
CAR	Acylcarnitine	LPC(12:0)	440.277717	184.007	[M+H]+	+
CE	Cholesterol ester	CE(17:0)	656.64392	369.35	[M+NH4]+	+
Cer	Ceramide	Cer(d18:1/4:0)	370.332119	264.2686	[M+H]+	+
DG	Diglycerides	DG(12:0/12:0)	474.425215	474.42521	[M+NH4]+	+
LPC	Lysophosphatidylcholine	LPC(12:0)	440.277717	184.007	[M+H]+	+
LPE	Lysophosphatidylethanolamine	LPE(14:0)	426.262064	285.254	[M+H]+	+
PC	Phosphatidylcholine	PC(13:0/13:0)	650.476082	184.007	[M+H]+	+
PE	Phosphatidylethanolamine	PE(12:0/12:0)	580.397832	439.39	[M+H]+	+
PG	Phosphatidylglycerol	PG(12:0/12:0)	628.418962	439.385	[M+NH4]+	+
PS	Phosphatidylserine	PS(14:0/14:0)	680.450261	257.2124	[M+H]+	+
SM	Sphingomyelin	PC(13:0/13:0)	650.476082	184.007	[M+H]+	+
TG	Triglycerides	TG(12:0/12:0/12:0)	656.582914	439.54	[M+NH4]+	+
PI	Phosphatidylinositol	PI(16:0/16:0)	809.525834	241.1	[M-H]-	-
FFA	Free fatty acids	FFA(16:0)-d31	286.47498	286.475	[M-H]-	-
Eicosanoid	Eicosanoid	5S-HETE-d8	327.28992	115.0036	[M-H]-	-

Table S3. The correlation between lipid metabolites and plasma biochemical indicators from clinical patient samples.

Class	Biochemical Indicators	PE			TG		
		name	r	*p*	name	r	*p*
Beneficial markers	APOA	PE(P-40:4)	0.3110	8.005×10^{-20}	TG(18:0/18:1/18:2)	-0.1382	7.265×10^{-5}
		PE(P-34:0)	0.2628	2.103×10^{-14}	TG(14:1/16:1/22:1)	-0.1379	7.495×10^{-5}
	HDLC	PE(P-40:4)	0.3216	6.459×10^{-25}	TG(14:1/16:1/22:1)	-0.1852	5.525×10^{-9}
		PE(P-34:0)	0.3069	9.790×10^{-23}	TG(14:1/16:0/22:2)	-0.1664	1.702×10^{-7}
Inflammation markers	ALT	PE(P-42:6)	-0.1408	2.593×10^{-5}	TG(14:0/20:1/22:3)	0.1250	0.0002
		PE(P-42:5)	-0.1400	2.866×10^{-5}	TG(14:0/20:1/20:3)	0.1158	0.0006
	AST	PE(P-38:5)	-0.1518	1.633×10^{-6}	TG(14:0/20:1/22:3)	0.0894	0.0049
		PE(P-34:3)	-0.1454	4.420×10^{-6}	TG(14:0/20:1/22:2)	0.0769	0.0156
Obesity risk indicators	TRIG	PE(P-44:7)	-0.0858	0.0079	TG(14:0/20:1/22:1)	0.8055	2.608×10^{-221}
		PE(P-44:6)	-0.0718	0.0257	TG(18:0/18:2/20:0)	0.7955	6.162×10^{-212}
	BMI	PE(P-44:5)	-0.1407	3.234×10^{-5}	TG(14:0/20:1/20:3)	0.1302	0.0001
		PE(P-44:6)	-0.1194	0.0004	TG(14:0/20:4/22:2)	0.1283	0.0002

Table S4. The correlation between lipid metabolites and plasma biochemical indicators from clinical patient samples.

**Lipid metabolite**	**Phenotype**	**r**	***p***	**Lipid metabolite**	**Phenotype**	**r**	***p***
TG	Body weight	0.4870	0.0294	PE	Body weight	-0.4184	0.0664
	Fasting glucose	0.6972	0.0006		Fasting glucose	-0.7428	0.0002
	iBAT weight	0.8402	<0.0001		iBAT weight	-0.8635	<0.0001
	iWAT weight	0.8413	<0.0001		iWAT weight	-0.8648	<0.0001
	Liver cholestrol	0.7990	<0.0001		Liver cholestrol	-0.7424	0.0002
	Liver Il1b	0.7517	0.0001		Liver Il1b	-0.8344	<0.0001
	Liver to body weight ratio	0.5615	0.0100		Liver to body weight ratio	-0.5862	0.0066
	Liver triglyceride	0.7389	0.0002		Liver triglyceride	-0.7781	0.0001
	Liver weight	0.5234	0.0179		Liver weight	-0.5028	0.0239
	Plasma ALT	0.6550	0.0017		Plasma ALT	-0.6573	0.0016
	Plasma AST	0.4826	0.0312		Plasma AST	-0.4705	0.0363
	Plasma cholestrol	0.7633	0.0001		Plasma cholestrol	-0.7523	0.0001
	Plasma triglyceride	0.7114	0.0004		Plasma triglyceride	-0.8147	<0.0001

Supplementary Figures

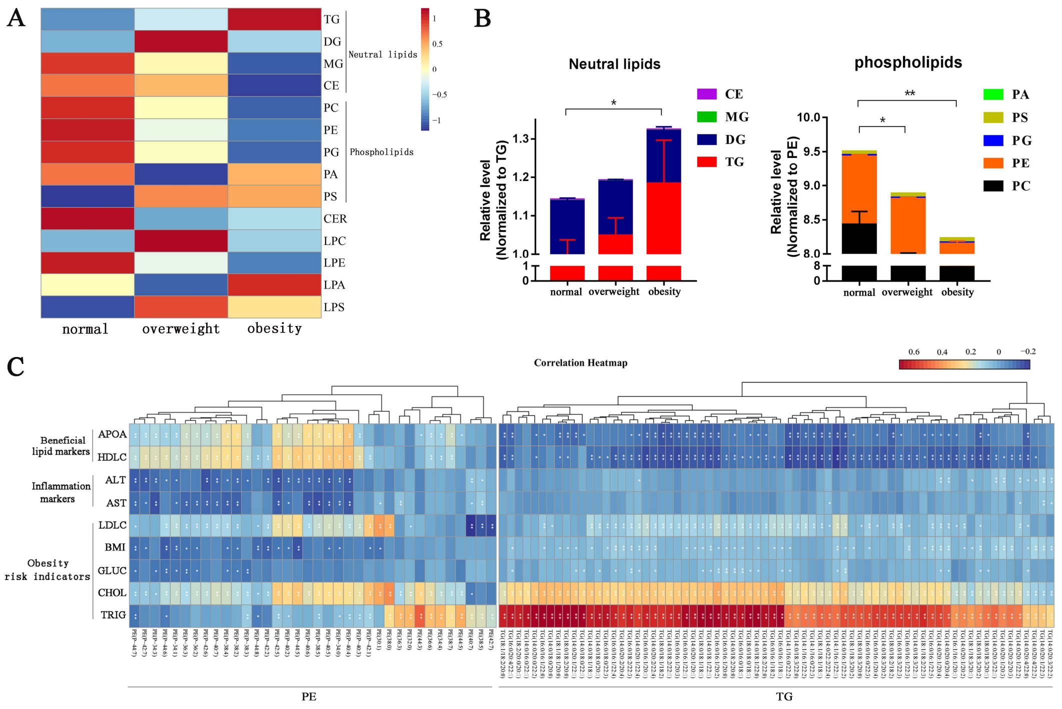

Figure S1. Lipidomic analyses from patient plasma samples. (A) Heatmap of relative mean levels of lipid species in patient plasma samples. (B) Neutral lipid analysis normalized to the TG in the normal group (left), and phospholipid analysis normalized to the PE in the normal group (right). *p<0.05; **p<0.01. (C) The correlation between lipid metabolites and plasma biochemical indicators from clinical patient samples. *p<0.05; **p<0.01. APOA, apolipoprotein A; HDLC, high-density lipoprotein cholesterol; ALT, alanine aminotransferase; AST, aspartate aminotransferase; LDLC, low-density lipoprotein cholesterol; BMI, body mass index; GLUC, blood glucose; CHOL, cholesterol; TRIG, triglyceride.

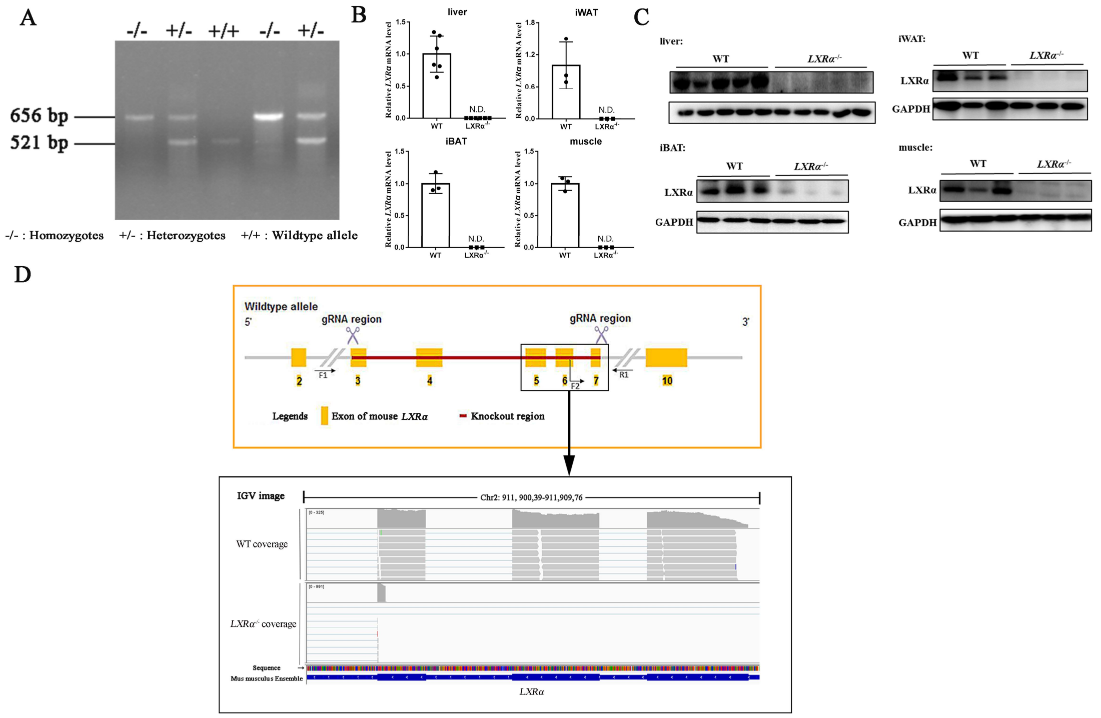

Figure S2. Genotype identification of *LXRα*-knockout mice. (A) PCR-based genotyping of wild-type (WT, +/+), heterozygotes (+/-), and *LXRα*-knockout (*LXRα*$^{-/-}$, -/-) mice. The bands of 521-bp and 656-bp represent WT and mutant alleles, respectively. (B) RT-qPCR analyses of *LXRα* mRNA expression in the liver, iWAT, iBAT and muscle from WT and *LXRα*$^{-/-}$ mice. Data are presented as mean ± SD. N.D., not detected. (C) Western blotting of LXRα protein in liver, iWAT, iBAT and muscle in WT and *LXRα*$^{-/-}$ mice. (D) Genotyping strategy and IGV visualization of high-throughput sequencing results between WT and *LXRα*$^{-/-}$ mice.

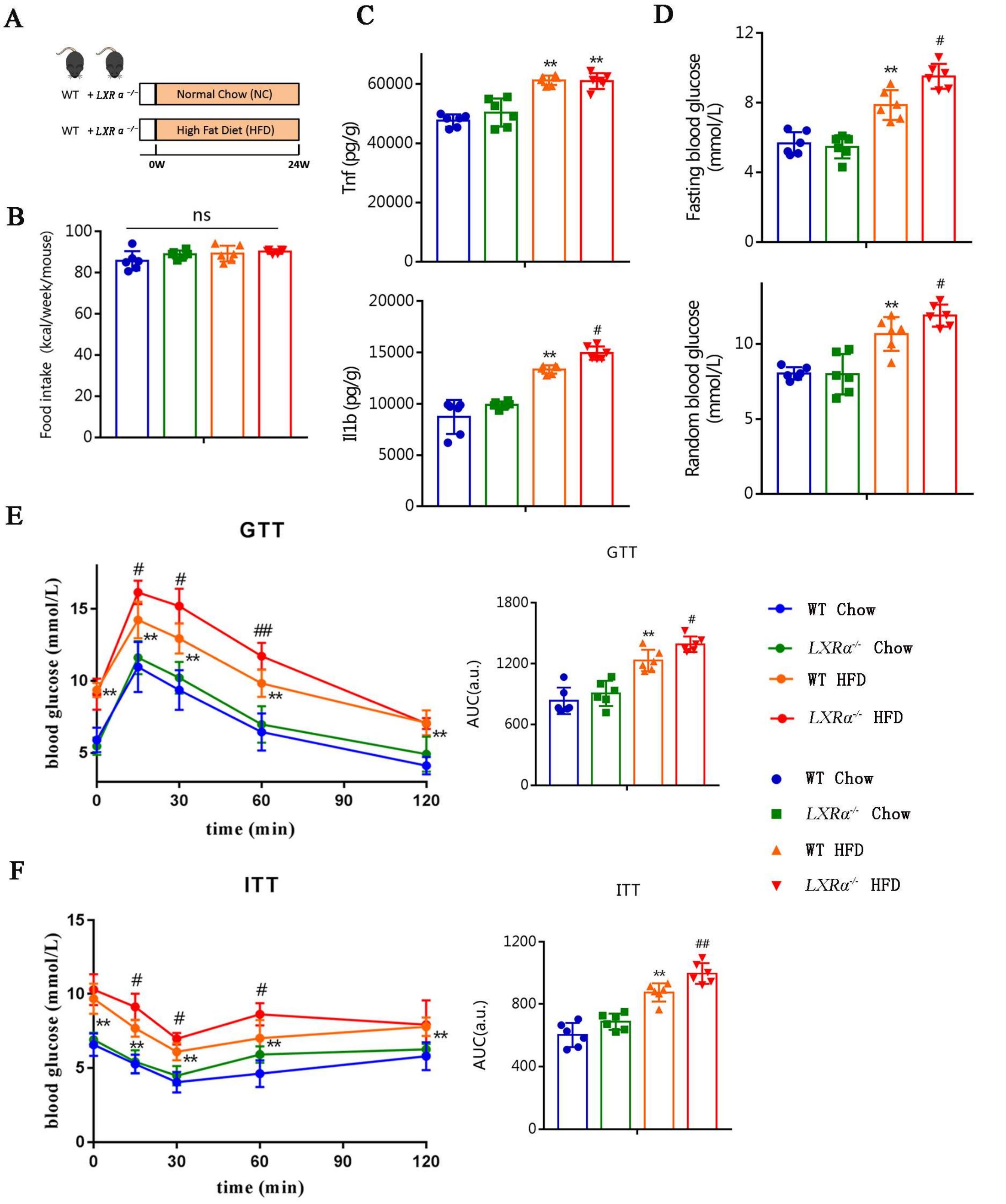

Figure S3. Biochemical and inflammation parameters between WT and *LXRα*$^{-/-}$ mice induced by normal chow (Chow) or high fat diet (HFD). (A) Schedule of the animal experiment. WT and *LXRα*$^{-/-}$ mice were fed normal chow (NC) or high-fat diet (HFD) for 24 weeks and randomly assigned to 4 groups: WT Chow, *LXRα*$^{-/-}$ Chow, WT HFD, *LXRα*$^{-/-}$ HFD. (B) The average food intake during week 24. (C) Tnf or Il1b production (measured by ELISA) in the livers of WT and *LXRα*$^{-/-}$ mice induced by NC or HFD. (D) Fasting blood glucose and random blood glucose of WT and *LXRα*$^{-/-}$ mice induced by NC or HFD. (E-F) Glucose tolerance test (GTT) (E) and insulin tolerance test (ITT) (F) of WT and LXRα $^{-/-}$ mice induced by NC or HFD. The inserts show the quantitation of AUC (area under the curve) values. Data were expressed as mean ± SD

(n=6). *p<0.05 and **p<0.01 compared to WT Chow; # p<0.05 and ## p<0.01 compared to WT HFD.

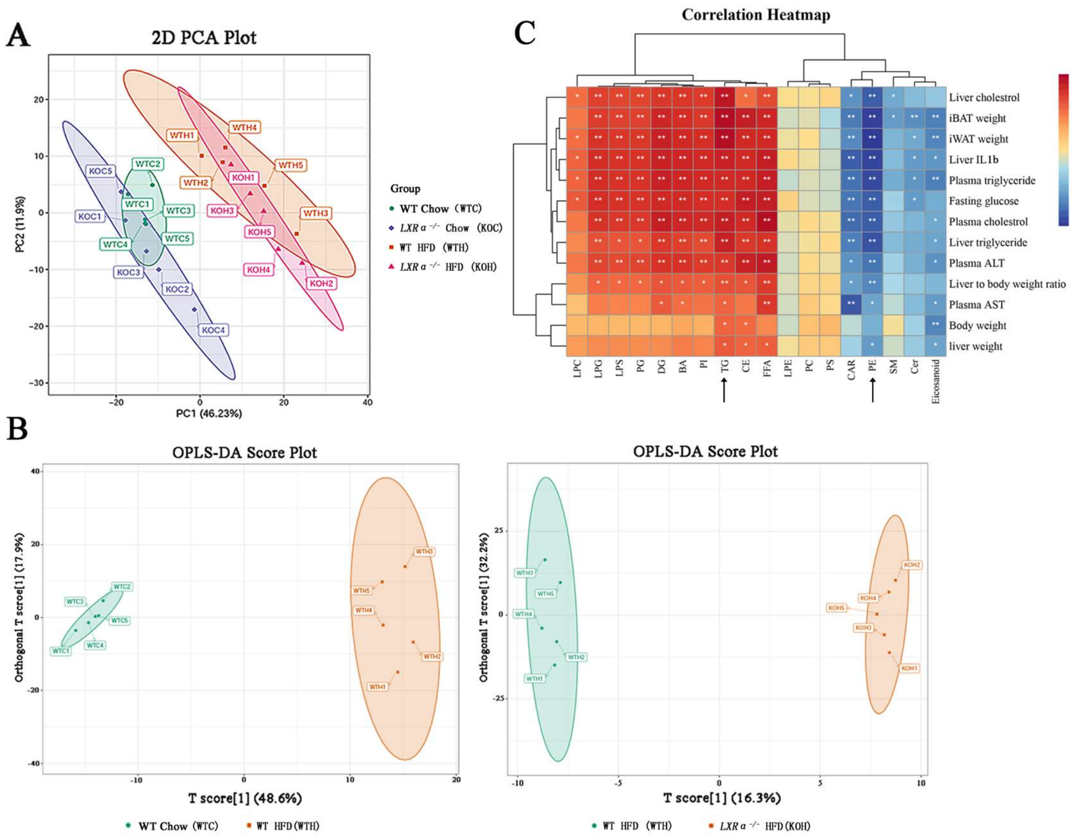

Figure S4. Lipidomic analyses of WT and *LXRα*$^{-/-}$ mice fed with Chow or HFD. (A) Principal component analysis (PCA) score plot of WT and *LXRα*$^{-/-}$ mice induced by NC or HFD. (B) Orthogonal Partial Least Squares Discrimination Analysis (OPLS-DA) score map for WT Chow and WT HFD (left), and for WT HFD and *LXR*$^{-/-}$ HFD (right), indicating high cohesion within groups and good separation among groups. (C) The correlation between lipid classes and mice phenotypes. *p<0.05; **p<0.01.

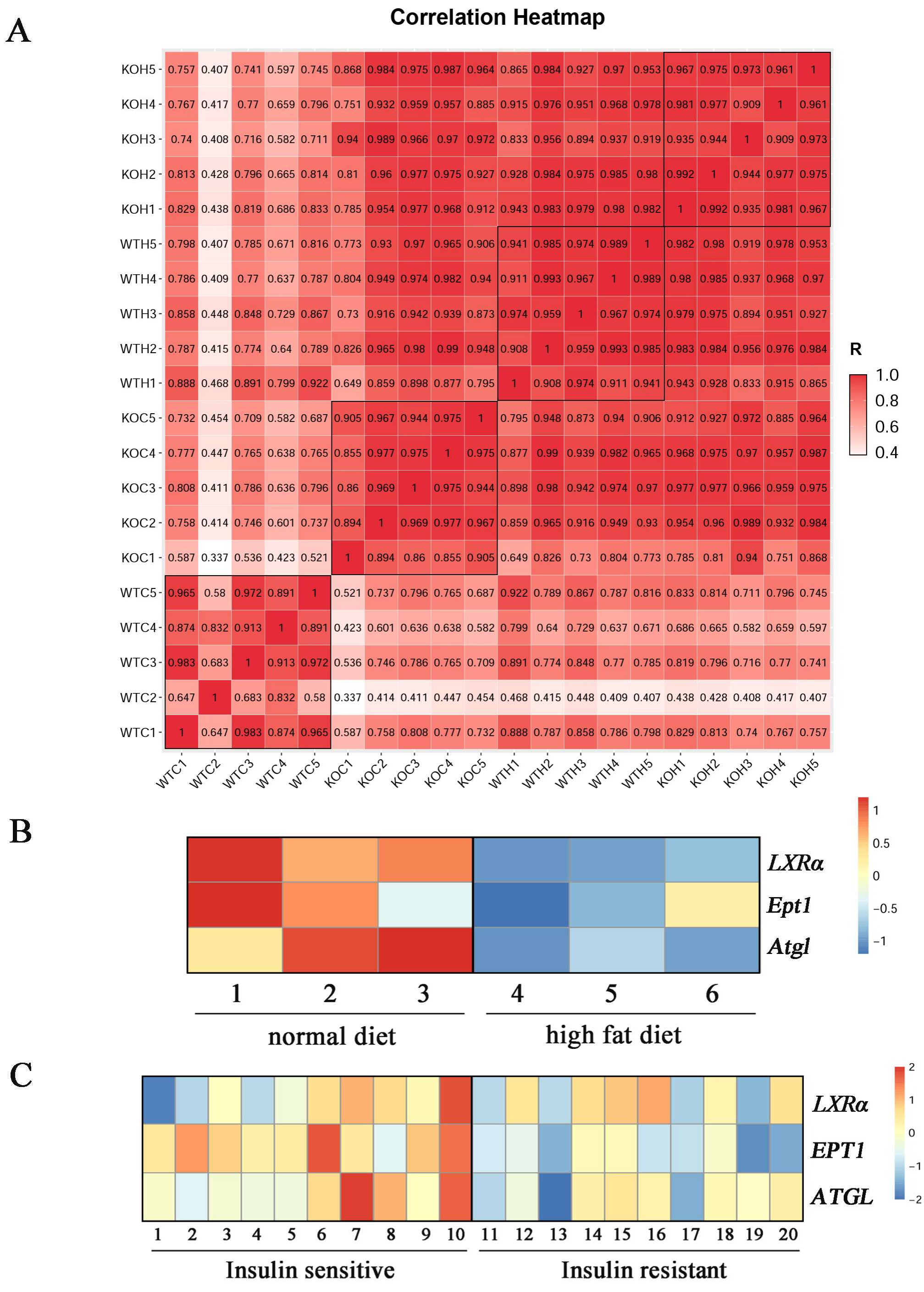

Figure S5. Decrease of *Atgl* and *Ept1* is an important downstream mechanism of lipid disorder in HFD-induced obesity. (A) The Pearson correlation of gene expression among samples. High correlation indicated good biological reproducibility. (B) Heatmap of gene expression in liver of mice fed with normal diet and high fat diet from GEO Profiles database (GDS4830). (C) Heatmap of gene expression in the subcutaneous adipose tissue of clinical patients with insulin sensitivity or resistance from GEO Profiles database (GDS3781).

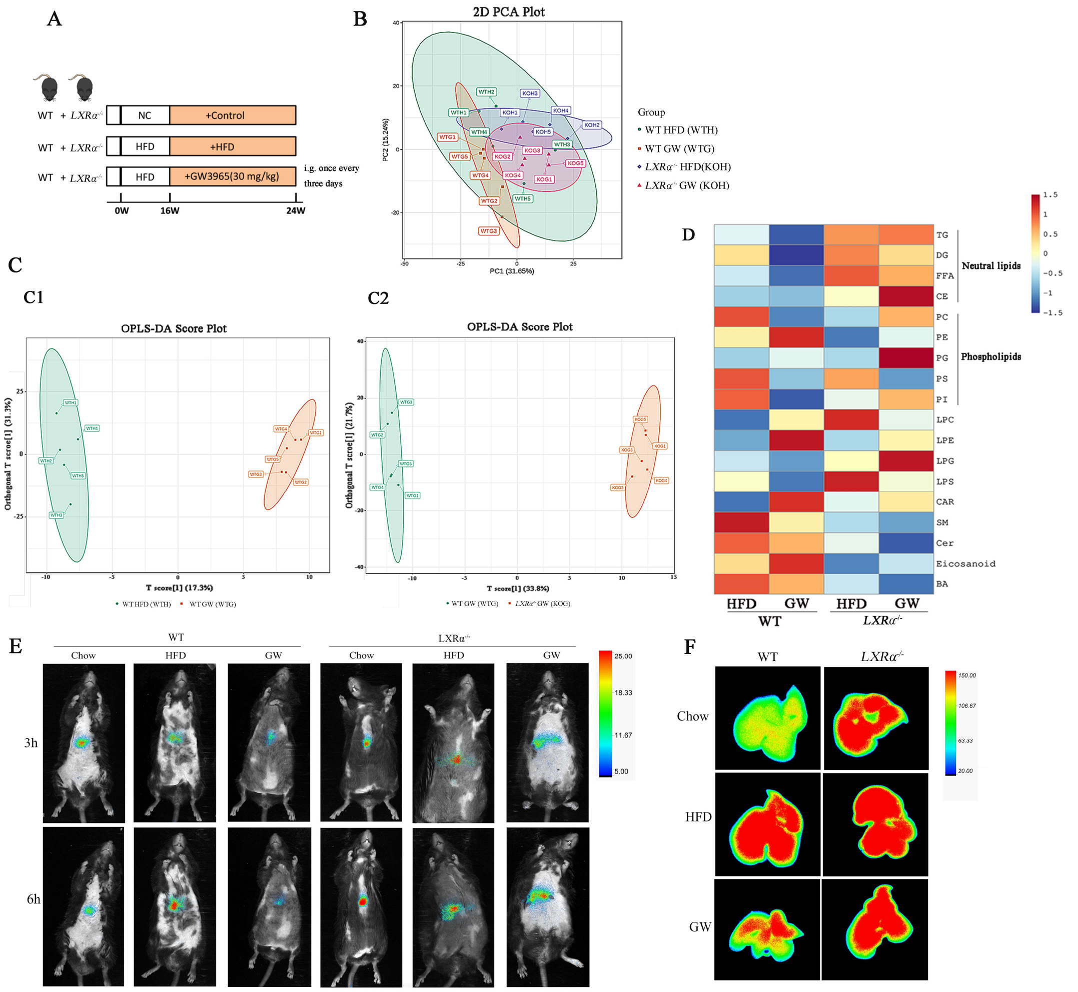

Figure S6. LXRα plays an important role in maintaining lipid homeostasis by affecting TG metabolism and PE synthesis. (A) Schedule of the animal experiment. WT and *LXRα*$^{-/-}$ mice were fed NC or HFD for 24 weeks, and GW3965 was administrated once every three days from week 16 to week 24 by gavage (n = 6). (B) PCA score plot of WT and LXRα$^{-/-}$ mice induced by HFD and treated with GW3965 or not. (C) OPLS-DA score map for WT HFD and WT GW (C1), and for WT GW and *LXRα*$^{-/-}$ GW (C2), indicating high cohesion within groups and good separation among groups. (D) Heatmap of average levels of lipid species concentrations in liver tissues of WT and LXRα$^{-/-}$ mice induced by HFD and treated with GW3965 or not. (E) Representative in vivo imaging system images of mice after tail vein injection of cy7-cholesterol. (F) Representative images of cy7-cholesterol fluorescence intensity in liver.

A

JASPAR
LXRE Motif

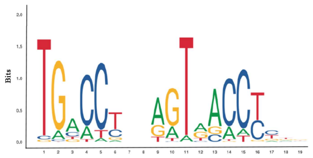

TFBS
Prediction

Score	Promoter	Relative score	Predicted sequence
Nr1h3::Rxra	EPT1	10.4605	TGATCGCAAGTAATCCCTC
		8.79138	TAAACTTCAGTGTCCTTTAC
	ATGL	13.2914	TGACCTCAGGTGATCTGCC
		8.34436	GGTCCAGAGATGACCTCAG
		8.21798	TGACTTAAAGCGACTCCTT
		8.15718	TGACCAGGAGAAAAATGCG
		7.45023	TCCCCCTAGATGGCCCCAT
		7.06447	TGGCCTATTTTTAAATTTT
		6.70696	TGACCTCGTGATCCACTCA

B

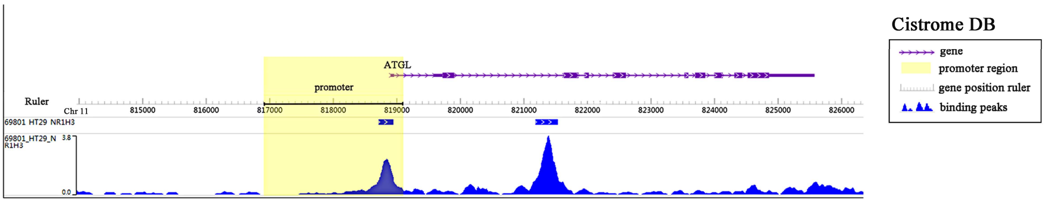

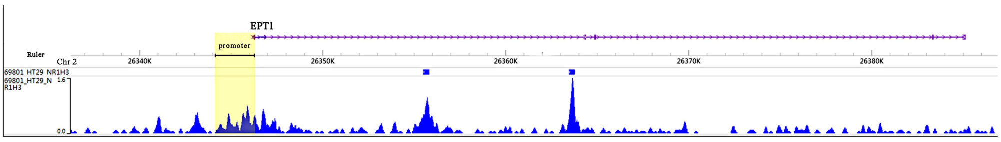

Figure S7. LXRα regulates the transcription of *EPT1* and *ATGL*. (A) JASPAR predicts the transcription factor binding site (TFBS) information of LXRα protein binding to the *ATGL* and *EPT1* promoter. (B) ChIP-seq data from Cistrome DB indicating enrichment of LXRα protein to ATGL and EPT1 promoter (Cistrome DB ID: 69801, GEO ID: GSM2042848).

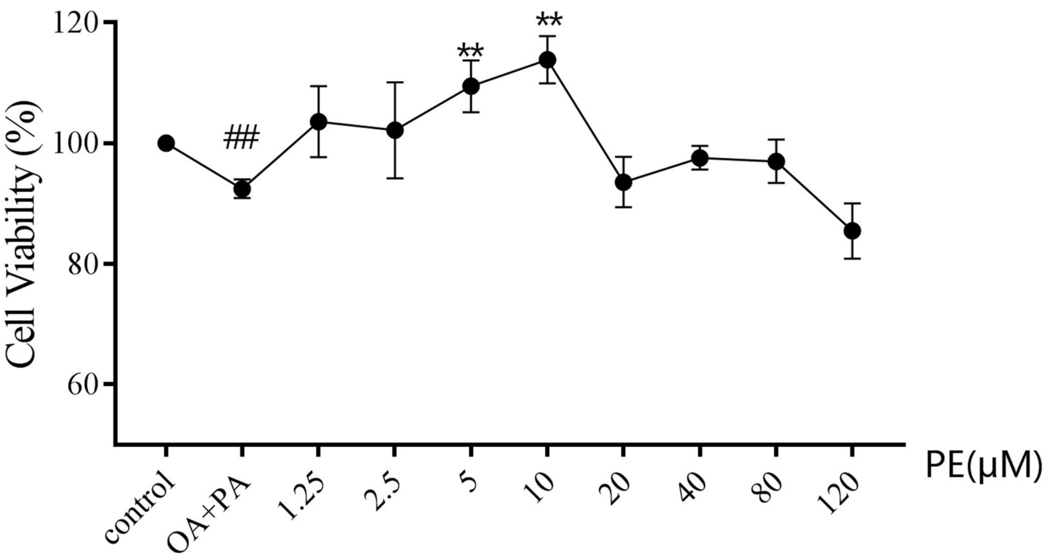

Figure S8. Evaluation of the dose-dependent cytotoxicity effects of PE in primary hepatocytes from *LXRα*$^{-/-}$ mice after treated with oleic acid (OA, 500 μM) and palmitic acid (PA, 250 μM). Data are mean ± SD (n = 5). # indicates a significant difference between the control group and the OA+PA group; $^{\#}p < 0.05$, $^{\#\#}p < 0.01$. * indicates a significant difference between the OA+PA group and the group treated with PE; $^{*}p < 0.05$, $^{**}p < 0.01$.

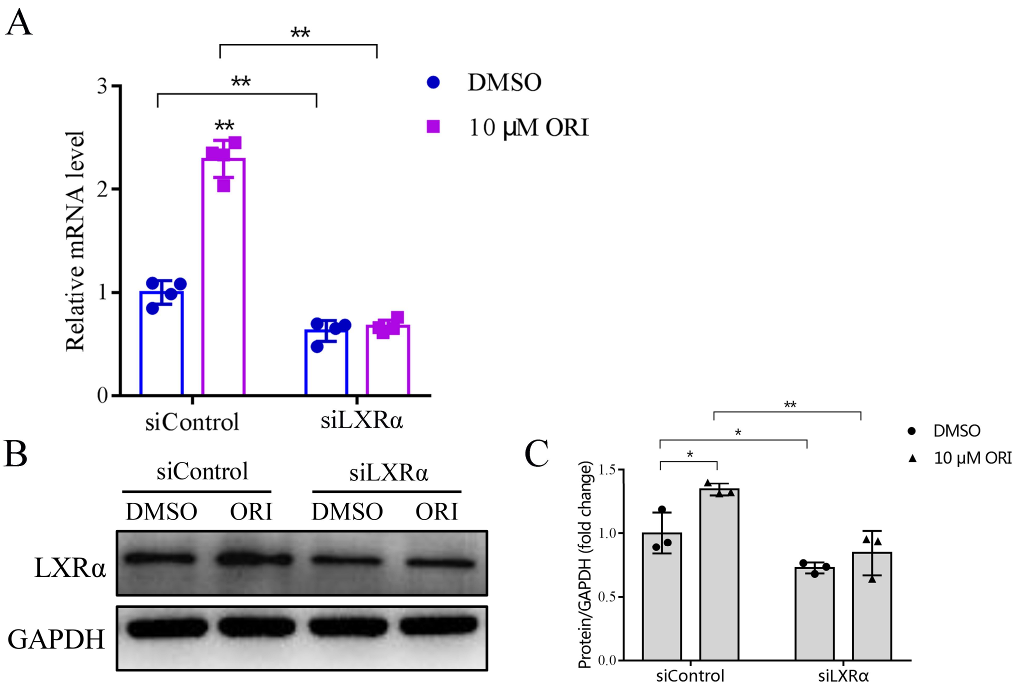

Figure S9. The identification of oridonin (ORI) as a LXRα inducer. (A-C) LXRα mRNA (n=4) and protein (n=3) levels in HepG2 cells treated with 10 μM oridonin (ORI) for 48 h after transfection with siLXRα or siControl. Data are mean ± SD. **p*<0.05; ***p*<0.01.

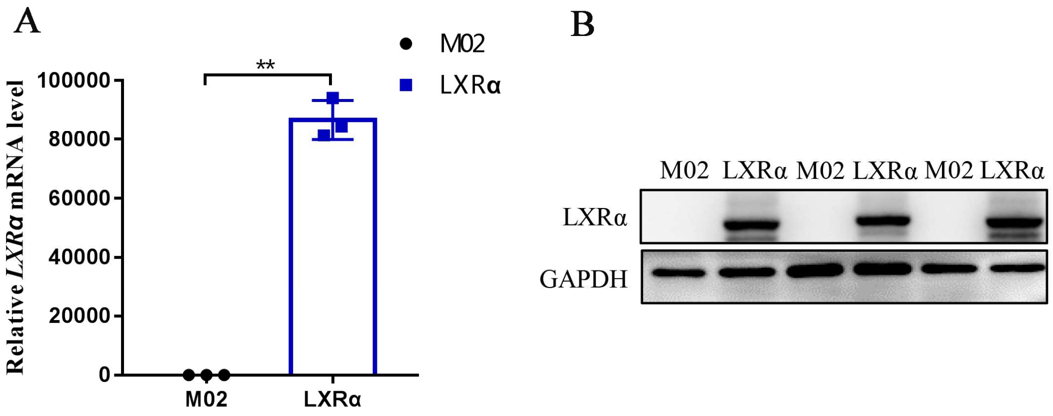

Figure S10. Model validation of LXRα overexpression on primary hepatocyte from *LXRα*$^{-/-}$ mice. (A-B) LXRα mRNA and protein expression in primary hepatocyte of LXRα$^{-/-}$ mice transfected with M02 or LXRα expression plasmid for 48 h. Data are mean ± SD (n=3). ***p*<0.01.
