# Supplementary material for: Restoration of lipid homeostasis between TG and PE by the LXRα-ATGL/EPT1 axis ameliorates hepatosteatosis
Source: Cell Death Dis. 2023 Feb 6;14(2):85. doi: 10.1038/s41419-023-05613-6 (PMC9902534; doi:10.1038/s41419-023-05613-6)

Supplementary Materials for

Restoration of lipid homeostasis between TG and PE by the LXRα-ATGL/EPT1 axis ameliorates obesity

Yulian Chen[1#], Huanguo Jiang[1#], Zhikun Zhan[1#], Jindi Lu[1], Tanwei Gu[1], Ping Yu[1], Weimin Liang[1], Xi Zhang[1], Shuwen Liu[1], Huichang Bi[1], Shilong Zhong[1 2] and Lan Tang[1*]*

*Corresponding Author Address: NMPA Key Laboratory for Research and Evaluation of Drug Metabolism, Guangdong Provincial Key Laboratory of New Drug Screening, School of Pharmaceutical Sciences, Southern Medical University, Guangzhou 510515, China. Tel. /fax: +86 20-61648596. Email: Email: Lan Tang, tl405@smu.edu.cn; Shilong Zhong, zhongsl@hotmail.com.

Full unedited gel for Figure 2:

p-p65 antibody (1:1000 dilution, 65kDa,3033T, CST, USA)

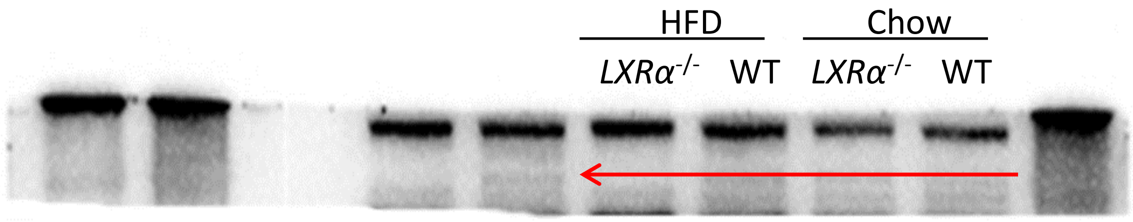

p65 antibody (1:1000 dilution, 65kDa,8242T, CST, USA)

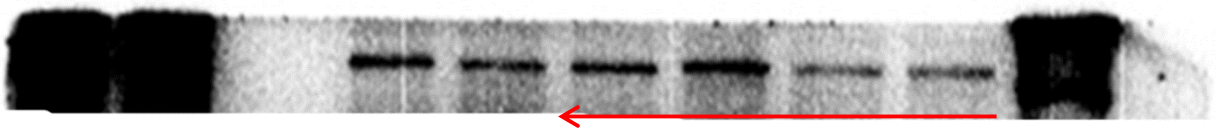

GAPDH antibody (1:3000 dilution, ab8345, Abcam, USA)

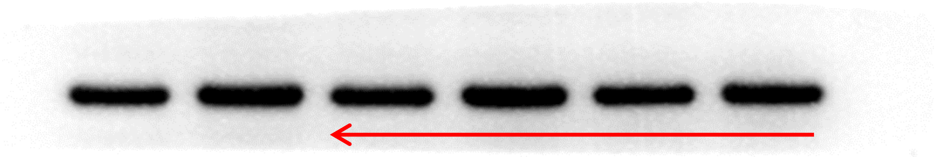

Full unedited gel for Figure S2:

LXR*α* antibody (1:500 dilution, ab41902, Abcam, USA)

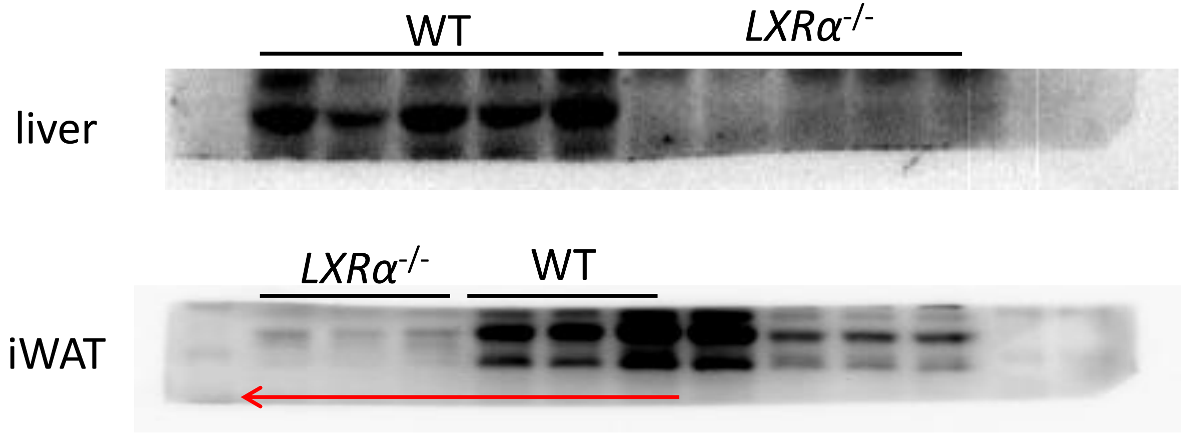

GAPDH antibody (1:3000 dilution, ab8345)

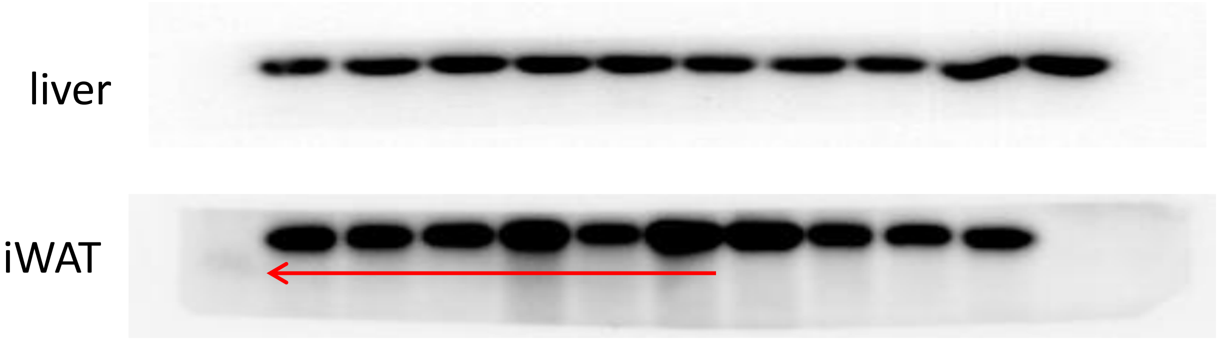

Full unedited gel for Figure S2:

LXR*α* antibody (1:500 dilution, ab41902, Abcam, USA)

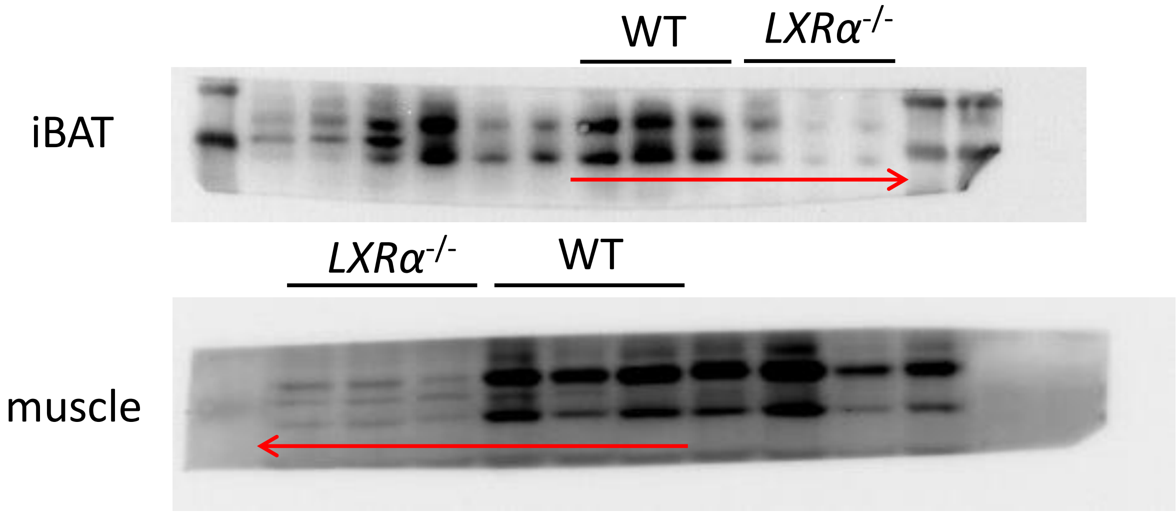

GAPDH antibody (1:3000 dilution, ab8345)

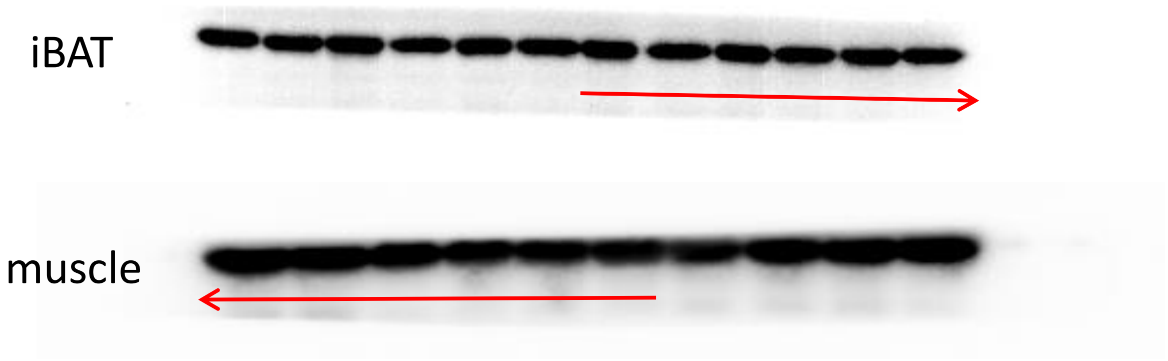

Full unedited gel for Figure 4:

LXR*α* antibody (1:500 dilution, ab41902, Abcam, USA)

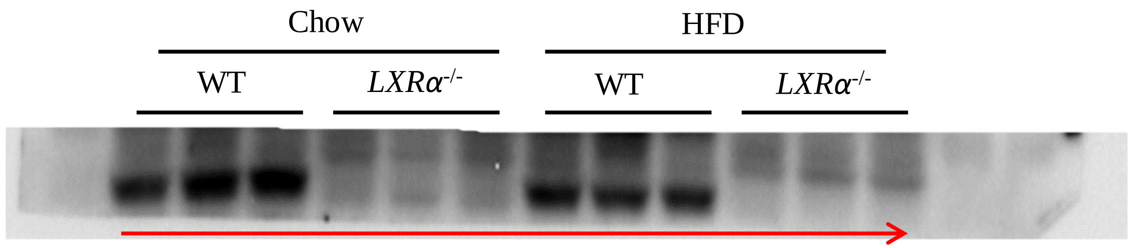

ATGL antibody (1:500 dilution, DF7756, Affinity, China)

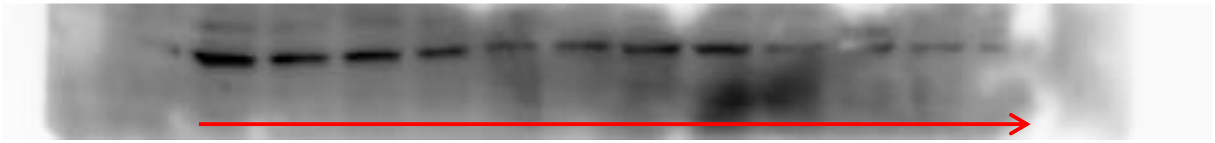

EPT1 antibody (1:250 dilution, ab194554)

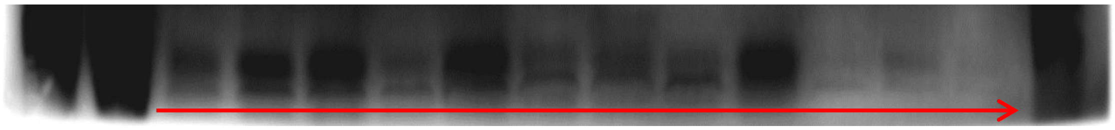

GAPDH antibody (1:3000 dilution, ab8345)

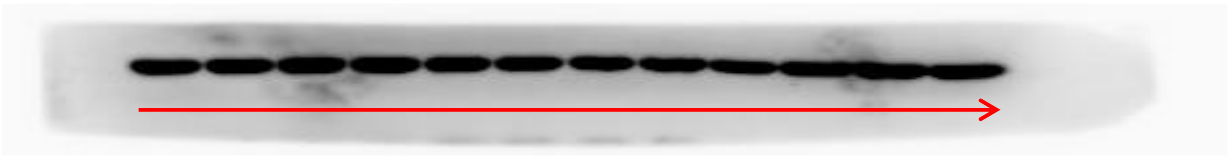

Full unedited gel for Figure 5:

LXRα antibody (1:500 dilution, ab41902, Abcam, USA)

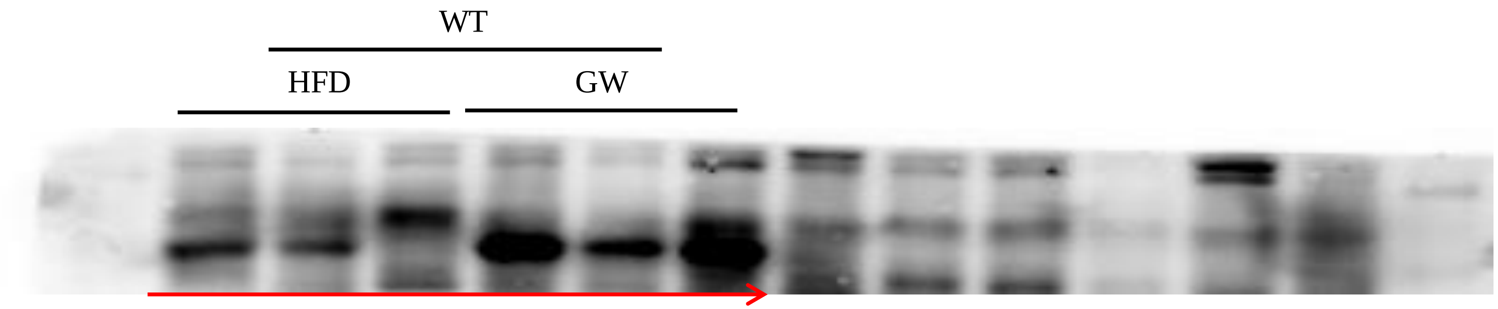

ATGL antibody (1:500 dilution, DF7756, Affinity, China)

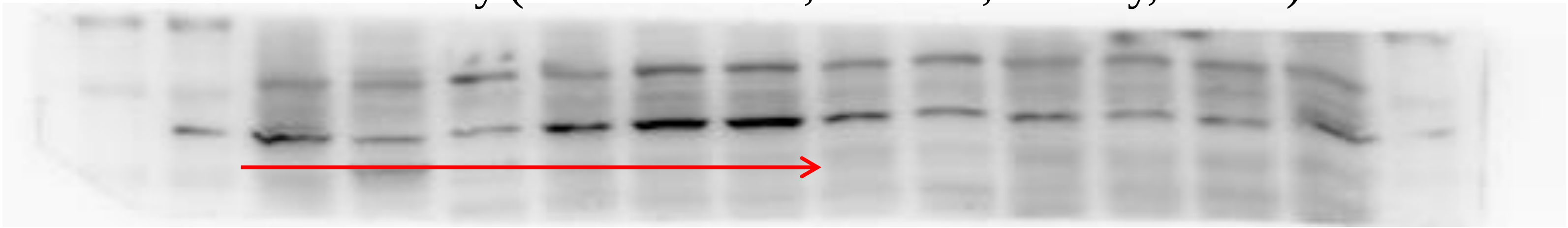

EPT1 antibody (1:250 dilution, ab194554)

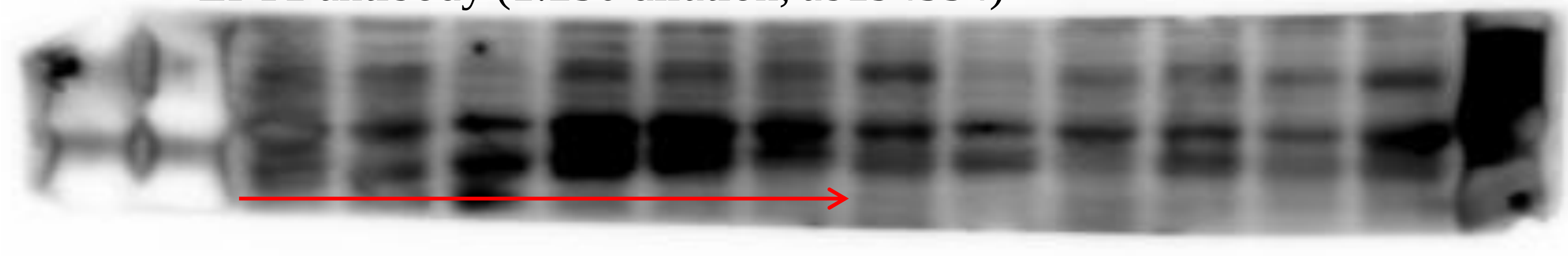

GAPDH antibody (1:3000 dilution, ab8345)

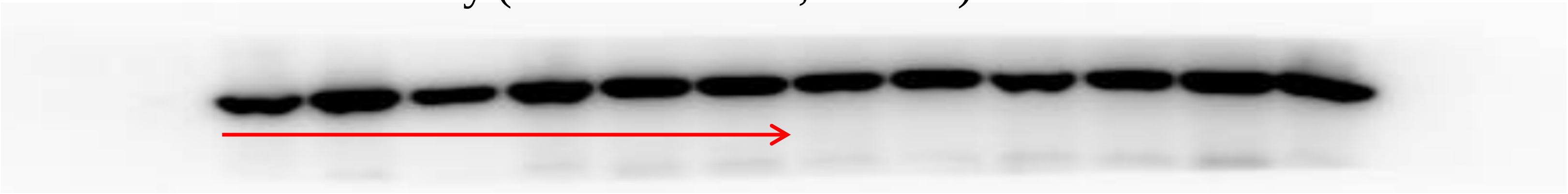

Full unedited gel for Figure 6B:

LXRα antibody (1:500 dilution, ab176323, Abcam, USA)

siLXRα control 10 20 40 80 (nM)

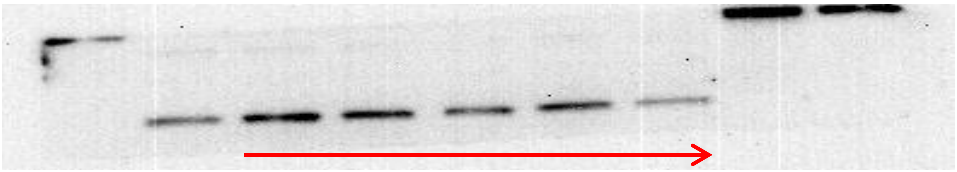

ATGL antibody (1:500 dilution, DF7756, Affinity, China)

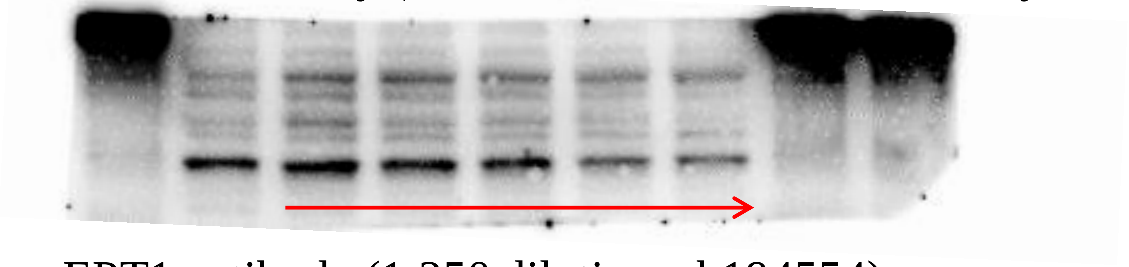

EPT1 antibody (1:250 dilution, ab194554)

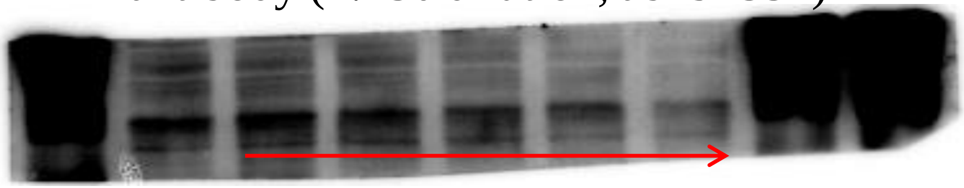

GAPDH antibody (1:3000 dilution, ab8345)

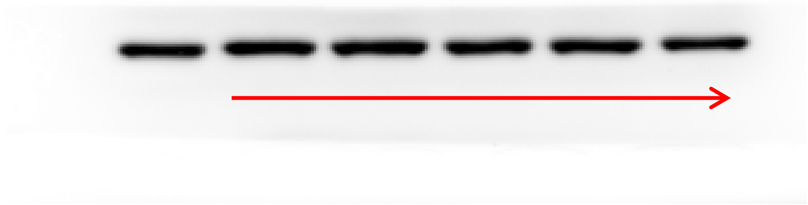

Full unedited gel for Figure 6D:

LXR*α* antibody (1:500 dilution, ab176323, Abcam, USA)

GW 0 2.5 5 (mM)

LXR*α*

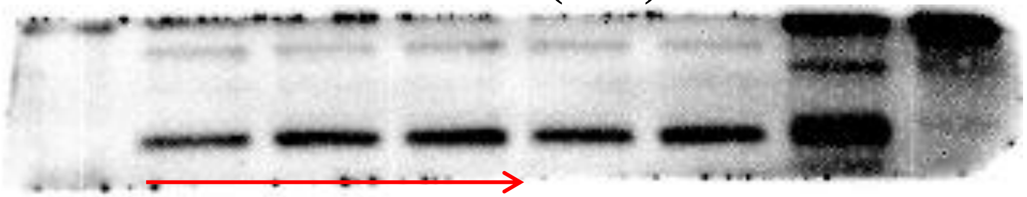

ATGL antibody (1:500 dilution, DF7756, Affinity, China)

ATGL

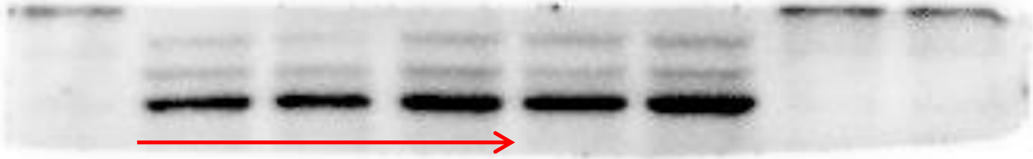

GAPDH antibody (1:3000 dilution, ab8345)

GAPDH

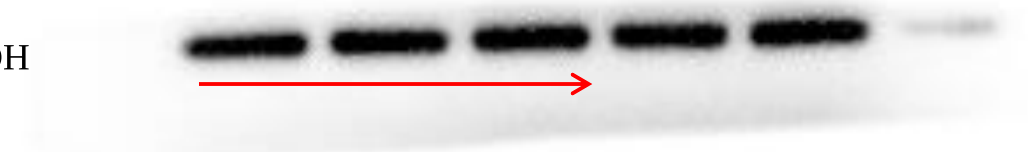

EPT1 antibody (1:250 dilution, ab194554)

GW 0 2.5 5 (μM)

EPT1

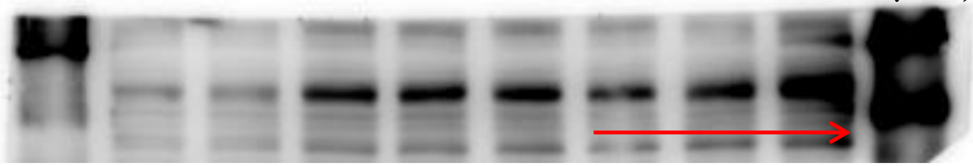

GAPDH antibody (1:3000 dilution, ab8345)

GAPDH

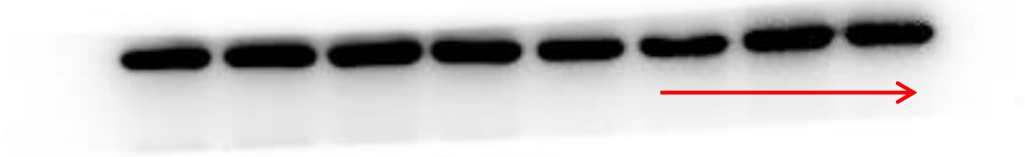

Full unedited gel for Figure 8:

LXRα antibody (1:500 dilution, ab176323, Abcam, USA)

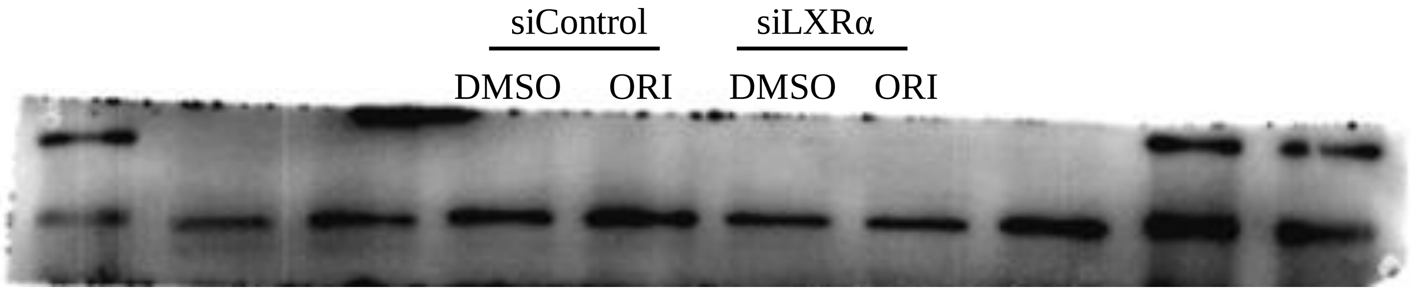

GAPDH antibody (1:3000 dilution, ab8345)

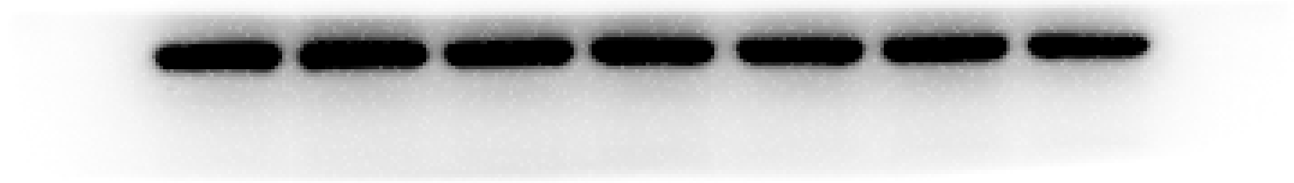

Full unedited gel for Figure S10:

LXRα antibody (1:500 dilution, ab41902, Abcam, USA)

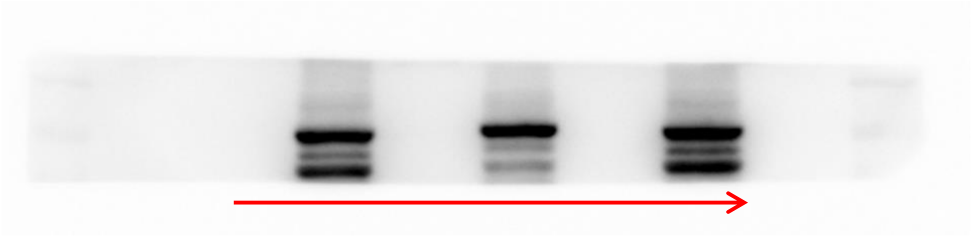

GAPDH antibody (1:3000 dilution, ab8345)

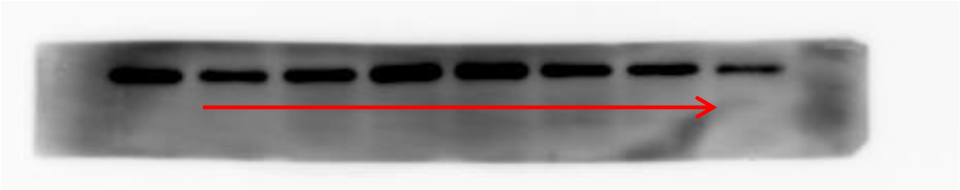

Supplement: Supplementary file 2 — Original Data File [file 41419_2023_5613_MOESM2_ESM.pdf]
